# Highly Efficient Wideband Microwave Absorbers Based on Zero-Valent Fe@*γ*-Fe_2_O_3_ and Fe/Co/Ni Carbon-Protected Alloy Nanoparticles Supported on Reduced Graphene Oxide

**DOI:** 10.3390/nano9091196

**Published:** 2019-08-25

**Authors:** Francisco Mederos-Henry, Julien Mahin, Benoit P. Pichon, Marinela M. Dîrtu, Yann Garcia, Arnaud Delcorte, Christian Bailly, Isabelle Huynen, Sophie Hermans

**Affiliations:** 1 Institute of Condensed Matter and Nanosciences (IMCN), Division of Molecules, Solids and Reactivity (MOST), Place Louis Pasteur 1, Université catholique de Louvain, B-1348 Louvain-la-Neuve, Belgium; 2 Department of Chemical Engineering and Biotechnology, University of Cambridge, Phillipa Fawcett Drive, West Cambridge Site, Cambridge CB3 0AS, UK; 3 Institut de Physique et Chimie des Matériaux de Strasbourg, CNRS, Université de Strasbourg, UMR 7504, F-67000 Strasbourg, France; 4 Faculty of Electrical Engineering and Computer Science & MANSiD Research Center, Stefan cel Mare University, 720229 Suceava, Romania; 5 Institute of Condensed Matter and Nanosciences (IMCN), Division of Bio and Soft Matter (BSMA), Croix du Sud 1, Université catholique de Louvain, B-1348 Louvain-la-Neuve, Belgium; 6 Institute of Information and Communication Technologies, Electronics and Applied Mathematics (ICTEAM), Place du Levant 3, Université catholique de Louvain, B-1348 Louvain-la-Neuve, Belgium

**Keywords:** magnetic nanoparticles, reduced graphene oxide, nanocomposites, microwave absorbing materials, electromagnetic interference shielding

## Abstract

Electronic systems and telecommunication devices based on low-power microwaves, ranging from 2 to 40 GHz, have massively developed in the last decades. Their extensive use has contributed to the emergence of diverse electromagnetic interference (EMI) phenomena. Consequently, EMI shielding has become a ubiquitous necessity and, in certain countries, a legal requirement. Broadband absorption is considered the only convincing EMI shielding solution when the complete disappearance of the unwanted microwave is required. In this study, a new type of microwave absorber materials (MAMs) based on reduced graphene oxide (rGO) decorated with zero-valent Fe@*γ*-Fe_2_O_3_ and Fe/Co/Ni carbon-protected alloy nanoparticles (NPs) were synthesized using the Pechini sol-gel method. Synthetic parameters were varied to determine their influence on the deposited NPs size and spatial distribution. The deposited superparamagnetic nanoparticles were found to induce a ferromagnetic resonance (FMR) absorption process in all cases. Furthermore, a direct relationship between the nanocomposites’ natural FMR frequency and their composition-dependent saturation magnetization (*M_s_*) was established. Finally, the microwave absorption efficiency (0.4 MHz to 20 GHz) of these new materials was found to range from 60% to 100%, depending on the nature of the metallic particles grafted onto rGO.

## 1. Introduction

Electronic systems and telecommunication devices based on gigahertz electromagnetic (EM) waves have been massively developed in the last decades. Having become ubiquitous, the extensive use of these media has contributed to the emergence of diverse electromagnetic interference (EMI) or pollution problems [1]. Furthermore, the so-called electromagnetic hypersensitivity (EHS) disorder has been attracting worldwide attention given its potential consequences on public health [2]. A large range of applications is concerned, from commercial and scientific electronic instruments to the whole current microwave-based telecommunication system and its related electronic devices. Considering the potential impact of such disruptive effects, an active quest for novel, highly efficient EMI shielding material solutions animates current scientific research in this area.

The classical EMI shielding solution is based on EM reflectors, such as metal foils or coatings (e.g., Faraday cages) [3]. However, these methods are being increasingly considered unsatisfactory given that they simply deflect the unwanted EM signal, shifting the problem elsewhere. EM shielding by absorption is a much better option as it radically solves the problem by completely removing the disruptive EM wave. 

Ferromagnetic resonance (FMR) is a physical phenomenon occurring in the microwave range that has already been exploited to design novel EMI shielding materials [4]. Indeed, an intense absorption process takes place at the FMR frequency, the value of which is partly determined by the composition of the FMR-active material. Typically, these materials are magnetically soft metals, such as Fe, Co, and Ni, their alloys, ferrites, and titanates [5].

When compared to other microwave absorbing materials (MAMs), metallic magnetic materials have high saturation magnetization (*M_s_*) and thus higher magnetic permeability (*μ*) so that they can be used to fabricate thinner composites with similar EMI shielding performance [4]. Furthermore, given that they are conductive, ferromagnetic metals and their alloys can increase the rate and width of absorption [5]. 

Magnetic nanoparticles (MNPs) have been deposited onto different nanocarbon supports (NcS), such as graphene [6,7], N-doped graphene [8,9], or graphene oxide [10], carbon nanotubes [11], or carbon nanofibers [12], for various applications, such as batteries or catalysis. Such nanocomposites have also been proposed as efficient EMI shielding materials [7,8,9]. Indeed, the MNPs allow adjusting the system’s magnetic permeability (*μ_r_*), while the nanocarbons’ electrical properties can be exploited to easily vary the system’s electrical permittivity (*ε_r_*), as we previously demonstrated [13]. Consequently, this synergic assembly of the MNPs and NcS individual EM features allow tuning an EMI shielding material’s absorption capabilities [14,15,16].

MNPs@NcS nanocomposites have been produced using a wide range of synthetic techniques. Wet chemical [17], solvothermal [17], hydrothermal [6], or inert atmosphere thermal [6] reduction processes, carbonization [12], direct [10], or electroless [7,11] plating and sol-gel [18] techniques are just a few of them. In this paper, we proposed a novel, Pechini-based preparative approach to obtain carbon-protected zero-valent iron (ZVI) and binary or ternary Fe/Co/Ni alloy nanoparticles (NPs) supported on reduced graphene oxide (rGO).

The Pechini method is a sol-gel synthesis technique starting from a polymerizable metal complex precursor. It was first described in the Pechini patent [19] within the context of alkaline earth titanates and niobates film deposition for the production of capacitors. The advantages of the Pechini method include its simplicity, the use of widely available reagents, and the obtaining of products with homogeneous characteristics. Therefore, it is nowadays commonly used for the synthesis of many materials, including laser gain materials [20], superconductors [21], perovskites [22], catalysts [23], and nanoparticles [24]. 

In the Pechini process, metal salts (usually nitrates) are dissolved alongside citric acid to form a metal citrate complex. In a second step, ethylene glycol is added and the solution is heated to induce a polyesterification reaction. This results in the formation of a complex-polyester gel with metallic atoms evenly distributed among the polymer matrix (see Supplementary Material (SM), Figure S1). The formation of this organic matrix during the first stages of the synthesis ensures that the complexed metals are evenly dispersed at high concentrations. The obtained gel is then calcined at high temperature, causing the pyrolysis of the polymer. Calcination produces NPs with homogeneous particle size, thanks to the homogeneity achieved in the precursor gel matrix [25]. Indeed, such a matrix ensures that small crystallites are evenly formed when nucleation occurs, tightly controlling particle size. Also, for binary or ternary systems, such as those addressed in this study, the gel matrix ensures that the different metals are effectively mixed at the atomic scale [25], ensuring the preparation of an end-material with homogeneous composition. 

The Pechini process is generally used to obtain oxides. However, it has been claimed that if the calcination step is carried out under inert or reducing atmosphere, metal@graphitic carbon core-shell nanoparticles (NPs) can be synthesized [18]. Such a shell would favor the long-term stability of ZVI or iron-containing alloy NPs, required by our intended applications. Indeed, it could function as a barrier for oxygen diffusion into the metallic or alloy core, effectively preventing the NPs oxidation [26]. For instance, the ZVI NPs can be readily oxidized into non-magnetic iron oxides, such as wustite (FeO) or hematite (*α*-Fe_2_O_3_) [27]. In such a case, the FMR-related EM absorption properties would be irremediably lost. 

The Pechini preparation of carbon-protected ZVI NPs@rGO has already been reported in the scientific literature [18]. The novelty of our contribution resides in the fact that we optimized several of the Pechini synthetic parameters to obtain a tighter control on the carbon-protected ZVI NPs composition, size, and spatial distribution over the graphenic sheets. More importantly, we showed for the first time that this synthetic method could be used to simply and straightforwardly synthetize rGO-supported binary and ternary Fe/Co/Ni alloy NPs with excellent control over their composition and size. This is particularly valuable for the ternary FeCoNi@rGO nanocomposite for which only two synthetic routes have been described in the scientific literature so far [7,28]. However, the proposed methodologies are either complex, requiring a series of painstaking synthetic steps [7], or offer a poor control over the composition of the obtained product, resulting in a mixture of several binary and ternary alloy phases [28]. Furthermore, the published EM characterization of similar Fe/Co/Ni NPs (free-standing or NcS-supported) [28,29,30,31] is usually restricted to a Jaumann configuration where frequency-selective absorbing performance is primarily controlled by the thickness of the sample. In our case, a novel coplanar transmission line-based technique, allowing to directly characterize the EM properties of the as-synthesized nanopowders [32,33], was used. Consequently, we could guarantee that the recorded microwave behaviors resulted exclusively from the studied nanocomposites. To the best of our knowledge, this is the first time that the wideband microwave absorption observed in these materials can be indubitably attributed to changes in the MNPs composition and the magnetic properties arising therefrom.

## 2. Materials and Methods 

### 2.1. Materials

Graphene oxide (GO) was purchased from Nanoinnova Technologies SL (Toledo, Spain). All other reactants were supplied by Across Organics (Merelbeke, Belgium), Alfa Aesar (Kandel, Germany), Merck (Overijse, Belgium), Sigma Aldrich (Darmstadt, Germany), or VWR (Leuven, Belgium) and engaged as received. More details on each reactant are provided in the Supplementary Materials (SM), Section S1.

### 2.2. Synthesis of Ferromagnetic Nanoparticles Supported on Graphene Oxide

The synthesis of ZVI@rGO nanocomposites was adapted from a Pechini-type method described in the scientific literature [18]. A typical synthesis is described below for a ZVI@rGO product with a 50% Fe/C mass loading rate. 

Fifty milligrams of GO, 180.8 mg of iron (III) nitrate nonahydrate (Fe(NO_3_)_3_·9H_2_O), 516 mg of citric acid (CA), and a magnetic stir bar were introduced in a small round-bottom flask. The ethanol at a volume of 3.3 mL was added, the flask was closed with a septum, and the mixture was stirred until complete dissolution of the reactants. The suspension was then sonicated at 80 W for 1 h (VWR ultrasound cleaner USC 1200-THD) to disperse GO. A total of 112 μL of ethylene glycol (EG) was added, and the suspension was heated with a reflux condenser in an oil bath at 90 °C for 3 h. The suspension was then poured in a porcelain combustion boat and placed in a drying oven under vacuum at 30 °C for 2 h. The obtained gel-covered ceramic plate was introduced in a tubular furnace (Carbolite Gero STF16, Hope Valley, UK) and heated to 750 °C for 2 h under H_2_/Ar 5/95 %v/v atmosphere. The temperature was increased and decreased at a rate of 1.7 °C/min. The recovered powder was stored in the air with no particular precautions.

The ratios 1:1.5 citric acid:ethylene glycol (CA:EG) and 6:1 citric acid:metal (CA:M) were used in the methodology described above. Nonetheless, we decided to test three other CA:EG (1:1, 1:1.5, 1:11) and two CA:M (3:1, 6:1) reactant ratios to determine the optimal proportions required to deposit NPs with homogeneous size and spatial distributions onto rGO.

Once the optimal synthetic conditions were determined, the same methodology described above for ZVI NPs was used to deposit FeCo, FeNi, and FeCoNi alloy NPs onto rGO. In all cases, the CA:EG ratio and CA:M ratios were of 1:1.5 and 6:1, respectively. Adapted quantities of Fe(NO_3_)_3_·9H_2_O, Co(NO_3_)_2_·6H_2_O, and Ni(NO_3_)_2_·6H_2_O salts were used as precursors to obtain the desired equimolar binary or ternary alloy compositions.

### 2.3. Characterization

For detailed characterization information, please refer to Section S2 of the Supplementary Material (SM) document.

## 3. Results

The targeted ferromagnetic resonance (FMR) phenomenon depends on the system’s magnetic properties, such as its saturation magnetization (*M_s_*). These characteristics are themselves dependent on compositional and morphological factors. Therefore, tight control on the deposited NPs chemical composition, size, and spatial distribution on nanocarbon surface is essential for our intended applications.

Consequently, we first determined the optimal Pechini reactant proportions required to deposit ZVI NPs with homogeneous size and spatial distributions onto a commercial GO, reduced to rGO in situ during the synthesis. The resulting nanocomposites chemical composition was then thoroughly studied. In a subsequent step, using the optimal conditions determined for the ZVI@rGO synthesis, three different Fe, Co, and/or Ni alloy NPs@rGO were produced and fully characterized. Finally, the nanocomposites magnetic properties were determined, and their interactions with microwaves studied. 

### 3.1. Optimization of the Citric Acid to Ethylene Glycol (CA:EG) and Metal Precursor (CA:M) Ratios

In the Pechini synthesis, the viscosity of the gelled precursor has a direct influence on the homogeneity of the end products [34]. Therefore, numerous investigations have attempted to identify the critical factors governing the gel viscosity [34,35,36,37]. Their results highlighted the crucial role played by the citric acid to ethylene glycol molar (CA:EG) ratio and the citric acid to metal molar (CA:M) ratio. They also showed that the gel viscosities were optimized when using molar CA:EG and CA:M ratios ranging from 1:1.5 to 1.5:1 and 1:1 to 6:1, respectively [34].

In this study, three different CA:EG (1:1, 1:1.5, 1:11) and two CA:M (3:1, 6:1) ratios were tested for the ZVI@rGO synthesis. TEM characterization of the obtained nanocomposites confirmed that these ratios strongly influenced the deposited ZVI NPs size and spatial distribution. 

As shown in Table 1, the smallest ZVI NPs possessing the narrowest size distributions were obtained by using the 6:1 CA:M ratio at lower CA:EG ratios. Also, lower CA:EG and CA:M ratios favored a more homogeneous and denser deposition of ZVI NPS onto the rGO surface (see SM, Figure S2).

Based on these results, we inferred that the different CA:EG and CA:M ratios were influencing the ZVI NPs size and spatial distribution by changing the length of the polyester chains that make up the gels as well as the spacing between them (see SM Figure S3, top). Indeed, when using excess quantities of EG (1:11 CA:EG ratio), the resulting chains were presumably shorter than when using equimolar EG and CA quantities. Furthermore, unreacted EG moieties diluted the system, spacing the polymerized chains apart. The shorter and more diluted chains distributed heterogeneously over the surface of rGO. When calcined in the tubular oven, they induced the formation of ZVI NPs in localized areas. Possibly because of thermal sintering effects, the heterogeneously deposited NPs also possessed a wider size distribution.

On the other hand, the reason why the ZVI NPs size and density were increased at lower CA:M ratios is illustrated in the Supplementary Material Figure S3, bottom. As the amount of CA moieties was decreased, the coordinated metallic ions were closer to each other in the polymer gel chains. When calcined, the ZVI NPs were thus deposited closer to each other and, eventually, sintered to form larger particles.

The Fe/C mass loading rate (%LR) of ZVI NPs deposited onto rGO was determined by TGA. When aiming at 50% ZVI NPs LR, the obtained loading rate varied from a minimum of 38% to a maximum of 49%. The obtained results showed that the %LR decreased as the amounts of EG and CA increased (see Table S1 in SM). This trend could be explained by considering the highly reductive atmosphere used in the tubular oven (e.g., H_2_/Ar (5/95 %v/v) gas stream at 750 °C). Under these conditions, residual carbon coming from EG or CA is expected to graphitize and deposit onto rGO [18]. Therefore, when using larger proportions of the different carbon-containing reactants, such as in the CA:EG 1:11 or the CA:M 6:1 ratios, larger amounts of graphitic carbon might be found in the end products. This additional carbon might artificially decrease the Fe/C %LR. 

Also, the iron precursor amount was varied, and the EG and CA quantities were adjusted to easily vary the wt. %LR. Nanocomposites with approximately 20%, 30%, 40%, and 60% ZVI NPs wt. %LRs were thus obtained straightforwardly (see corresponding TGA thermograms in SM, Figure S4).

### 3.2. Characterization of the Chemical Composition of the ZVI Nanoparticles Deposited onto rGO

**Figure 1:**
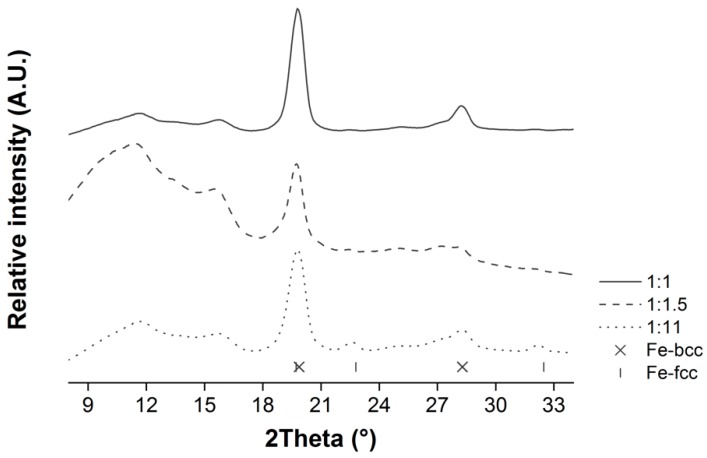
XRPD diffractograms of the different ZVI@rGO nanocomposites obtained at 1:1, 1:1.5, 1:11 CA:EG and a 6:1 CA:M ratios.

The ZVI@rGO nanocomposites were characterized by Powder X-ray diffraction (XRPD). As shown in Figure 1, the recorded spectra obtained with a molybdenum (Mo) anode showed a broad reflection around 12 °2θ (0.342 nm), which could be attributed to poorly ordered, short-range interactions between (0 0 2) planes of reduced graphene oxide (rGO) [37,38]. These graphitic reflections could be explained by the fact that GO is reduced to rGO under the H_2_/Ar (5/95 %v/v) atmosphere and high temperature used in our synthetic procedure (see **X**-ray photoelectron spectroscopy (XPS) results demonstrating the graphene sheets reduction in SM, Table S2, and Figure S5). Once reduced, rGO graphitic sheets interact via π-π stacking interactions similarly to graphite planes [39]. Moreover, graphitic deposits formed from CA or EG during the calcination step might also contribute to this signal.

As for the NPs, they seem to be composed of a mixture of cubic iron phases. Indeed, characteristic body-centered cubic (bcc) and face-centered cubic (fcc) reflections were detected (see Figure 1). Bcc reflections are the most intense, and, therefore, this crystalline phase is assumed to be the most abundant. fcc reflections are even less intense at the lower CA:EG ratios (1:1 and 1:1.5), independently of the CA:M ratio (not shown). A reflection around 16 °2θ (0.258 nm) suggested that maghemite (*γ*-Fe_2_O_3_) was also present in lesser amount. Nonetheless, its broadness might indicate that the iron oxide is poorly crystalline.

Mössbauer investigation of a ZVI@rGO nanocomposite synthesized using 1:1.5 CA:EG and 6:1 CA:M ratios also revealed three different iron species and allowed to quantify them. Indeed, as shown in Table 2 and Supplementary Material Figure S6a, two well-defined sextet patterns and a weak broad singlet were identified. The splitting of the first sextet (isomer shift (*δ*)= 0.11(1) mm/s) presented an internal field (*B_hf_*) value of ~34.1 T, which was consistent with Fe-bcc [40]. The second sextet (*δ* = 0.43(1) mm/s) presented a *B_hf_* ~39 T value, which was characteristic of maghemite [41], while the singlet, with an isomer shift of 0.05 mm/s, was typical of superparamagnetic Fe-fcc NPs [42]. Based on the quantification of assigned site-populations, the as-synthesized ZVI NPs seemed to be mainly composed of Fe-bcc (86%) and maghemite (10%), with the Fe-fcc phase accounting for the rest (4%). No evidence of other iron oxides was found in our data [43,44].

**Figure 2:**
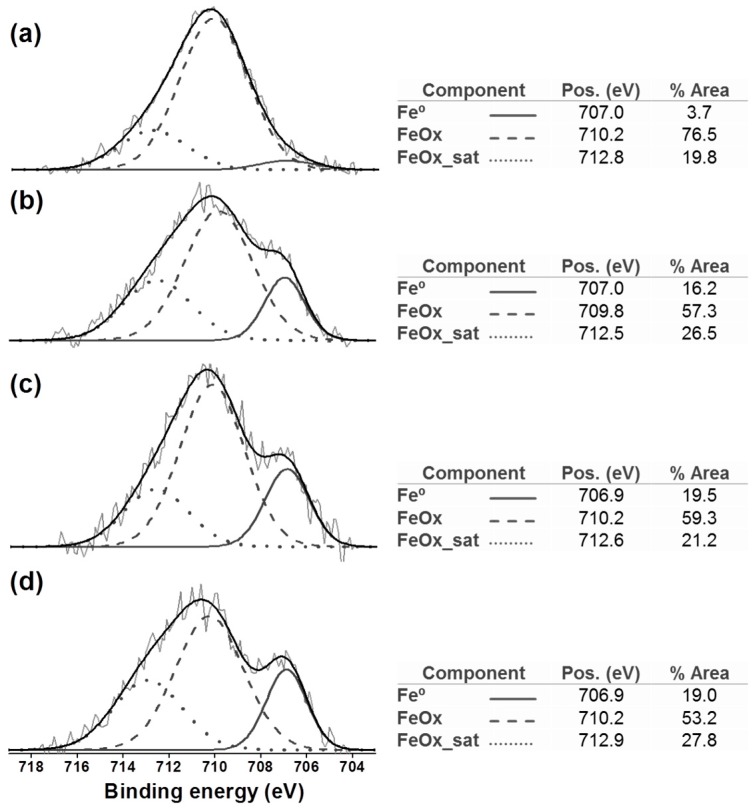
XPS Fe2p_3/2_ peak region showing metallic (Fe^0^) and oxidized (FeOx+FeOx_sat) components (comp.) in (**a**) ZVI, (**b**) FeCo, (**c**) FeNi, and (**d**) FeCoNi alloy NPs (nanoparticles) deposited on rGO (reduced graphene oxide).

XPS results confirmed the low iron oxide content. For instance, the decomposition of a ZVI@rGO nanocomposite O_1s_ peak region showed that oxygen bonded to organic carbon (C–org) was almost nine times more abundant than that bonded to iron (FeO*_x_*) (see SM, Figure S7). Interestingly, the decomposition of the Fe2p_3/2_ peak region showed a larger proportion of iron oxide as compared to metallic iron (Fe^0^) (see Figure 2). These results might indicate that the oxide, in this case, maghemite, is to be found on the surface of the deposited nanoparticles. Indeed, XPS is a surface-sensitive technique with a sampling depth of ~4.5 and 5 nm for iron and maghemite, respectively [45]. Therefore, the emitted photoelectrons would originate mostly from the outermost layer of the sampled NPs. If this layer is mainly composed of maghemite, the XPS spectra would show an artificially higher fraction of the FeO*_x_* component.

**Figure 3:**
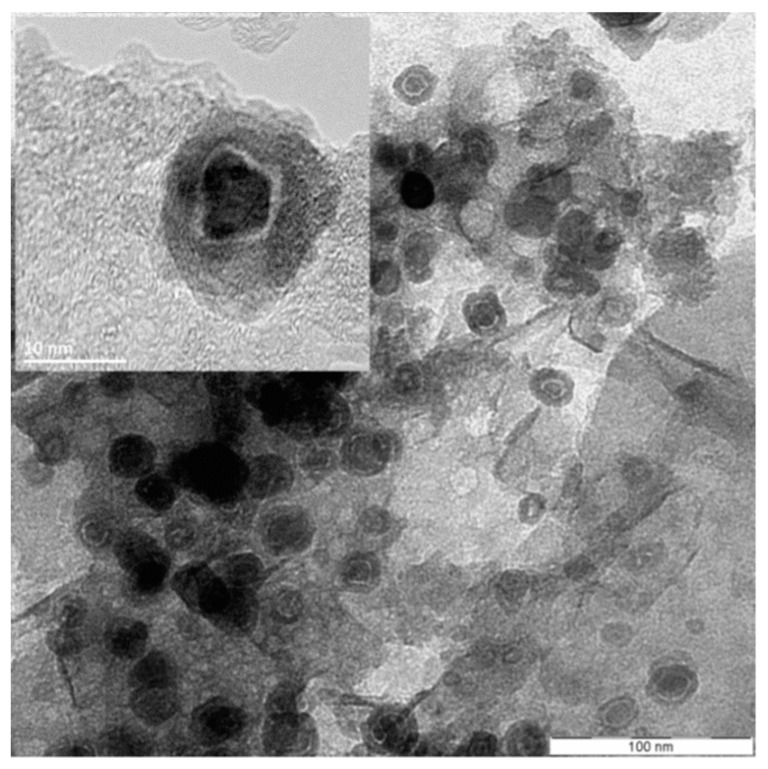
TEM and high-resolution TEM (HRTEM) (inset, upper left corner) images of a ZVI@rGO nanocomposite prepared with 1:1.5 CA:M and 3:1 CA:EG ratios. ZVI: zero-valent iron.

Confirmation of such a core-shell structure was obtained by transmission electron microscopy (TEM) and high-resolution TEM (HRTEM). Figure 3 shows representative TEM and HRTEM images in which the ZVI NPs are visualized with a darker core differentiated from a surrounding lighter shell layer. The outer shell has an irregular thickness with an average value of 3.1 ± 0.7 nm. Also, in some cases, a very light circle between the core and the shell was observed. This might be an empty space or local decohesion upon cooling due to the Fe^0^ core and the *γ*-Fe_2_O_3_ shell having different thermal expansion coefficients [46].

**Figure 4:**
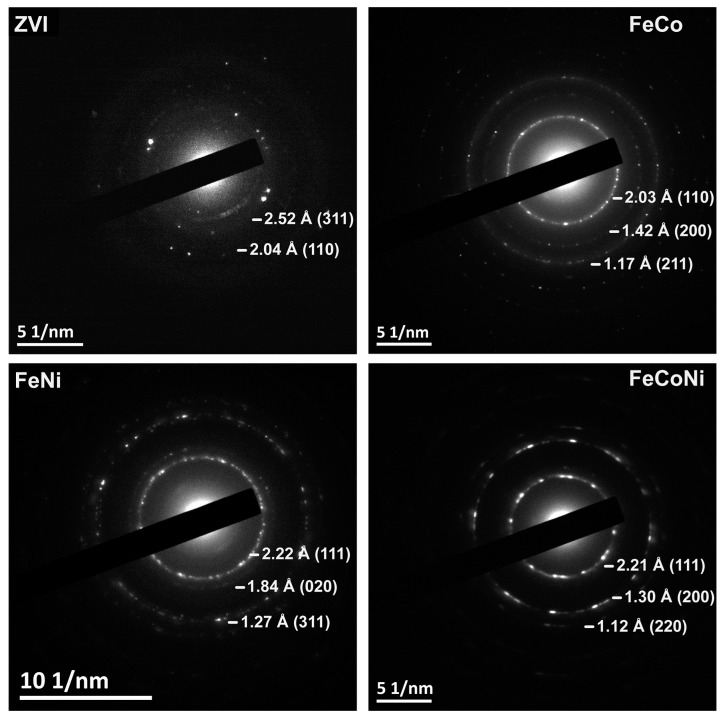
Electron diffractograms collected during HRTEM observation of the different nanocomposites. Calculated lattice spacing (in Angstroms, Å) and their corresponding (*hkl*) indices are included for each product.

In agreement with our XRPD, XPS, and Mössbauer results, lattice parameters calculated from HRTEM micrographs and electron diffraction patterns showed that the nanoparticles’ core is composed of metallic iron while maghemite is found in the shell [47] (see Figure 4). The presence of an iron oxide layer on the surface of ZVI NPs obtained by a similar synthetic methodology has previously been described [18]. However, to the best of our knowledge, this is the first time that the metal oxide phase is fully characterized and identified as maghemite. 

### 3.3. Synthesis and Characterization of Fe/Co/Ni Alloy NPs Deposited onto rGO

Based on the results obtained in the ZVI@rGO synthesis, 1:1.5 CA:EG and 6:1 CA:M ratios were chosen to perform the syntheses of Fe-containing alloy nanocomposites. Three types of equimolar binary (FeCo and FeNi) and ternary (FeCoNi) alloy NPs were deposited onto rGO using the same Pechini methodology. 

**Figure 5:**
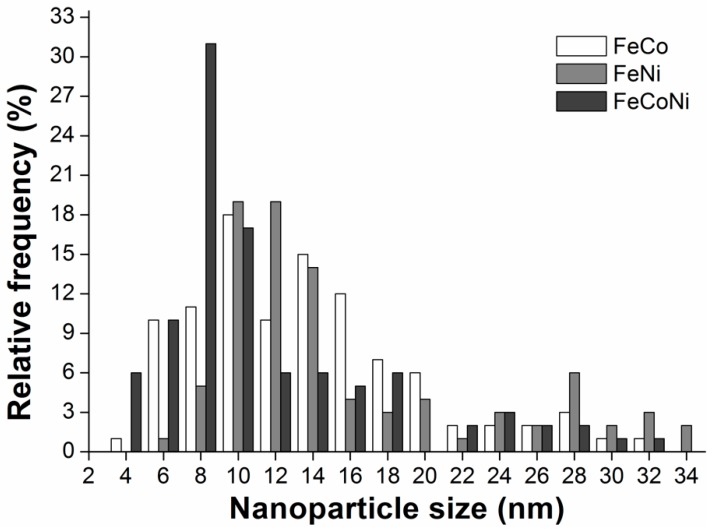
Alloy NPs size distribution histograms (from TEM images given in SM, Figure S6).

TEM observations were used to characterize the size distribution of alloy NPs deposited over the graphenic sheets. In contrast with what was observed for the ZVI NPs, all the alloy NPs size distributions were bimodal (see histograms in Figure 5 and TEM images in SM, Figure S8). Also, in all nanocomposites, the smaller NPs were more abundant than the larger ones. However, their particular average values varied slightly from one alloy to the other. For instance, in the FeNi and FeCo nanocomposites, the size of the smallest NPs was centered on 11 nm, while in the FeCoNi alloy, its average value was 8 nm. As for the larger NPs, central values around 25 nm were observed for both the FeCo and FeCoNi nanocomposites, being slightly higher (28 nm) for the FeNi products.

As in the ZVI nanocomposites, distinct core-shell structures were observed in all alloy samples (see SM, Figure S9). The observed shells also had an irregular thickness, but they were significantly thinner (2.3 ± 0.5 nm) than those observed in the ZVI NPs samples. Furthermore, they possessed a layered structure with an average ~3.30 Å layer interspacing value that is characteristic for graphite [48]. As mentioned before, obtaining a graphitic shell is important to ensure the long-term stability of the deposited NPs. Indeed, such a carbon shell is expected to limit oxygen diffusion into the metallic core, effectively preventing the NPs oxidation [26]. ToF-SIMS analysis of the alloy nanocomposites (see corresponding spectra in SM, Figure S10) also showed the presence of small amounts of binary metal oxides resulting from alloy oxidation, possibly at the surface of the NPs core.

ICP and point SEM-EDX analyses were performed to determine the alloy NPs composition. Results confirmed that the desired 1:1 or 1:1:1 elemental ratios were obtained for the binary and ternary alloys, respectively (see Table 3).

**Figure 6:**
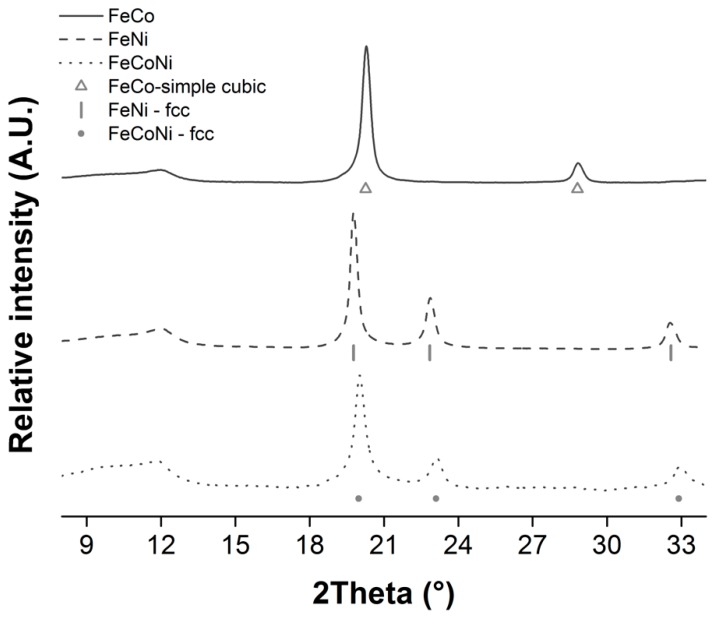
XRPD diffractograms of the different alloy nanocomposites obtained at 1:1.5 CA:EG and 6:1 CA:M ratios.

The three alloy nanocomposites were also characterized by XRPD. FeNi and FeCoNi NPs were found to be exclusively composed of fcc crystals. However, FeCo nanoparticles were composed of primitive (simple) cubic crystallites (see Figure 6). The obtained results were coherent with previous reports on these alloys’ crystallographic structures [49,50]. Lattice parameters calculated from HRTEM micrographs and electron diffraction patterns (see Figure 4) likewise confirmed that the desired alloy phases were obtained in all cases [51]. 

A deeper understanding of the different Fe alloy-nanocomposites structure and composition was gained by Mössbauer spectroscopy (see Table 2 and SM, Figure S6b–d). For instance, in the FeCo@rGO nanocomposite, a sextet (100% sites population) characterized by an isomer shift (*δ*) of 0.12(1) mm/s and an internal field (*B_hf_*) of ~34.6T was identified at 77K. Similar *B_hf_* and *δ* values have already been described for a FeCo alloy [52]. Furthermore, the *B_hf_* value indicated that the alloyed atoms were distributed in a relatively ordered manner. Indeed, *B_hf_* values larger than ~34.8T [53] denote increasing degrees of substitutional disorder in FeCo alloys, a completely disordered state being characterized by a *B_hf_* ~36T value [54].

In the FeNi@rGO product, a first magnetic sextuplet (approximately 70% sites population) at *δ* = 0.06(2) mm/s and distribution of hyperfine fields with *B_hf_* ~31.5T was observed. These values are typical for disordered FeNi alloys in the composition range 35−50% of Ni possessing an fcc arrangement [55,56]. A second sexted (24% population), with *δ* = 0.027(1) mm/s *B_hf_* ~34.1T was also identified. Such *δ* and *B_hf_* values are indicative of an ordered FeNi alloy with a lower percentage of Ni. Finally, a third signal (6%), with a *δ* = 0.16(1) mm/s value, was attributed to the presence of superparamagnetic FeNi NPs in the nanocomposite [55,57,58].

For the FeCoNi@rGO nanocomposite, a sextuplet (70% of sites population) with a *B_hf_* ~32T value was identified. A second sextuplet (30% of sites population) was found at *δ* = 0.35(1) mm/s with a *B_hf_* ~21.2 T. These values might indicate the presence of an fcc carbide phase, Fe_3_C, or Fe_4_C [42,59], which has already been identified at room temperature for similar materials [60]. 

XPS analyses of these nanocomposites clearly showed the presence of iron oxides (see Figure 2). However, the fact that such chemical species were neither detected by XRPD nor Mössbauer spectroscopy indicated that they were poorly crystalline and present in very limited quantities (less than 2%). As mentioned in Section 3.2, these results suggest that oxidized iron might be found on the surface of the alloy NPs. Nonetheless, when compared to their ZVI counterparts, the amount of oxidized iron was much lower in all iron alloy NPs (see Figure 2). This result showed that alloy NPs were more resistant to oxidation than ZVI NPs. This effect is documented in the scientific literature [61] and is explained by the higher reduction potential of cobalt (E^0^ Co/Co^2+^ = −0.28 V) and nickel (E^0^ Ni/Ni^2+^ = −0.27 V) as compared to iron (E^0^ Fe/Fe^2+^ = −0.44 V).

### 3.4. Characterization of the Nanocomposites Magnetic Properties and Microwave Absorption Behavior

The magnetic properties of the ZVI and Fe/Co/Ni alloy nanocomposites were studied by SQUID magnetometry. In all cases, magnetization curves recorded against the magnetic field in opposite directions did not overlap at 300 K, indicating a weak ferromagnetic behavior (see SM, Figure S11, top). 

Given that the sizes of our alloy nanoparticles were mostly below the superparamagnetic size limit (SPL, see Table 4), such a ferromagnetic behavior was unexpected. However, the presence of ferrimagnetic (FiM) *γ*-Fe_2_O_3_ or antiferromagnetic (AFM) Fe/Co/Ni oxides at the surface of the ZVI or alloy nanoparticles, respectively, might explain the presence of a coercive field at room temperature. Indeed, both types of oxides could increase the effective magnetic anisotropy energy of the deposited NPs. In the case of the *γ*-Fe_2_O_3_ shell layer, such an increase might be due to the presence of noncollinear or canting spin arrangements possessing a larger anisotropy than the core [62]. Consequently, an interfacial core-shell coupling, which increases the magnetic anisotropy energy of the whole NP, could be expected.

As for the surface AFM metal oxides, they were characterized by magnetic anisotropies much smaller than the ones of ferromagnetic (FM) metals. Thus, we expected some exchange bias (EB) coupling at the interface between the metal core and the oxidized shell. EB coupling consists of the pinning of interfacial spins in the AFM shell to the ones of the FM core [65], also resulting in extra magnetic anisotropy energy.

Table 4 shows the saturation magnetization (*M_s_*) obtained for the ZVI and alloy nanocomposites. The *M_s_* value for the ZVI@rGO nanocomposite fell between those found for similarly sized ZVI or maghemite NPs [64,65,66,67]. In this sense, it reflected the core-shell composition of the deposited ZVI NPs and the relative abundance of each phase, as discussed above. In the case of the different alloy nanocomposites, the *M_s_* decreased in the order FeCo > FeCoNi > FeNi. The observed trend showed that the *M_s_* increased as the amount of Fe and/or Co increased in the alloy NP composition, matching what has been observed for bulk metallic Fe (218 emu/g) > Co (161 emu/g) >> Ni (54 emu/g) *M_s_* values [68].

The fact that the obtained *M_s_* values were lower than those for the bulk ferromagnetic metals could be explained by the reduced sizes of the deposited NPs. Indeed, it is considered that NPs possess a disordered magnetic spin layer at their surfaces [69]. As the NPs size decreases, the disordered layer to NPs radius ratio becomes significant. Such surface spin disorder reduces the saturation magnetization for smaller nanoparticles [70].

As also shown in Table 4, the FeCo alloy possessed larger intrinsic coercivity (*H_c_*) and remanent magnetization (*M_r_*) values than the FeNi or FeCoNi alloys. This was due to the relative higher Co content found in the FeCo@rGO nanocomposite. Indeed, Co has the largest magnetocrystalline anisotropy of the three ferromagnetic metals and thus shows larger *H_c_* and *M_r_* values [71].

We also characterized the nanocomposites’ properties in the microwave range by measuring the scattering matrix and analyzing the scattering parameters representing an incoming microwave reflection (*S_11_*, *S_22_*) and transmission (*S_21_*, *S_12_*). The measurements were performed directly on the synthesized powders following a novel Vector Network Analyzer (VNA) methodology reported elsewhere [32,33]. Our method used a single coplanar transmission line test cell in which the powder was inserted, without any type of sample preparation. Consequently, we avoided sample thickness effects commonly observed in other EM characterization methodologies described in the scientific literature [29,30,31]. We could thus guarantee that the recorded microwave behaviors exclusively resulted from the studied nanocomposites.

Supplementary Material Figure S12, top, shows the results for the *S_21_* transmission parameter of a ZVI@rGO nanocomposite with an intended 50% Fe/C LR. A series of absorption processes were recorded, occurring even in the absence of an externally applied magnetic field. Also, their frequency shifted towards higher values as the external magnetic field was increased. 

All these experimental observations indicated that a ferromagnetic resonance (FMR) was occurring in the range of the microwave frequencies under study (40 MHz to 20 GHz). Indeed, according to Kittel [72], the resonance frequency of a ferromagnetic material is dependent on the strength of the applied static magnetic field, *H_z_*. Furthermore, if the deposited NPs are large enough to ensure a magnetic moment stability-lifetime, *τ*, larger than 10^−9^ s [73], an effective anisotropy field (*H_a_*) is induced in the sample. Under these conditions, a so-called natural resonance can be observed at zero static field (*H_z_* = 0). Thus, in perfect agreement with what was experimentally observed here, the Kittel FMR theory predicts the existence of natural resonance, at zero magnetic field, that tends to shift toward higher frequencies as *H_z_* is increased. 

Supplementary Material Figure S12, bottom, also shows that the incoming microwave is non-reciprocally transmitted through the nanocomposite, as evidenced by the non-zero signal resulting from the *S_21_*-*S_12_* parameter subtraction. This observation confirmed the ferromagnetic nature of the NPs deposited onto the nanocarbon substrate. Indeed, this behavior is expected when a coplanar waveguide, such as the one used in our EM characterization methodology, lies onto a ferromagnetic substrate [74].

**Figure 7:**
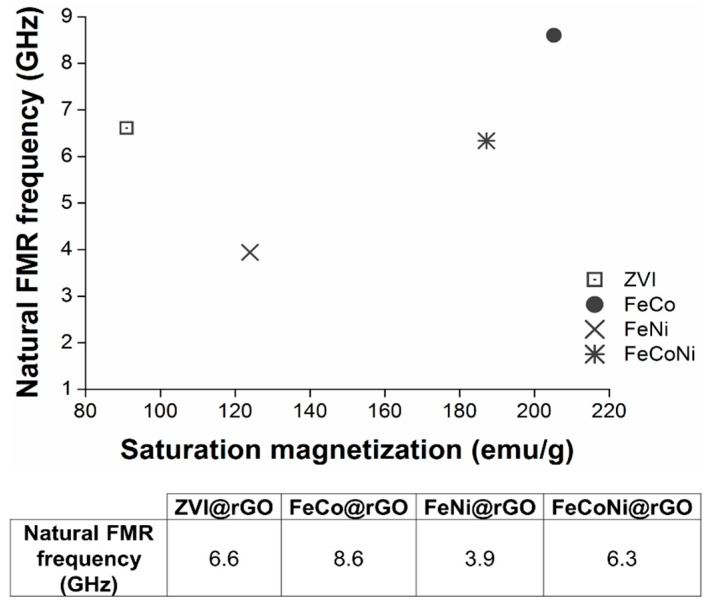
Dispersion relation between the different nanocomposites’ natural ferromagnetic resonance frequency and saturation magnetization.

Figure 7 shows the different nanocomposites’ natural FMR frequency plotted as a function of the saturation magnetization, *M_s_*. As shown in SM, Figure S13, the natural frequency was obtained by extrapolating, to zero applied magnetic field, the linear regression obtained from the resonant frequency versus applied magnetic field plots. For the alloy nanocomposites, the obtained results evidenced that their natural FMR frequency increased alongside their *M_s_*. These changes in *M_s_* were most probably due to the NPs different chemical composition. Indeed, the deposited NPs possessed similar average sizes. Likewise, they were mostly (hemi)spherical, and the nanocomposite flakes were randomly distributed in the powders, thus ruling out any anisotropic effect. In other terms, these results perfectly illustrated how variations in the NPs chemical composition induced a shift in the nanocomposites natural FMR frequencies. To the best of our knowledge, this is the first time that such a clear relationship between the NPs composition and their natural FMR resonances is determined for this type of nanocomposites. 

Interestingly, the ZVI@rGO nanocomposite did not follow this trend and showed a higher than expected natural FMR frequency. The recorded natural FMR frequency would rather correspond to the *M_s_* of similarly-sized ZVI NPs (e.g., 175 emu/g as extrapolated from the values reported by Kura et al. [66]). This observation suggested that the *γ*-Fe_2_O_3_ shell was not contributing to the FMR absorption process, which was solely due to the *α*-Fe core (Fe-bcc).

The FMR absorption at zero applied magnetic field of an *α*-Fe@*γ*-Fe_2_O_3_ core@shell nanoparticle was numerically simulated using a chain matrix formalism, described elsewhere [75,76]. The simulation aimed at determining the relative contribution of the core and the shell to the natural FMR frequency of the ZVI@rGO nanocomposite. The obtained results, shown in SM Figure S14, agreed with those observed experimentally. They also showed that the natural FMR frequency was mainly due to the ZVI core, given the thin maghemite shell’s contribution was extremely weak. Indeed, given maghemite’s *M_s_* value was three times inferior to that of *α*-Fe, the imaginary component of relative magnetic permeability, responsible for the FMR absorption, was thrice as low too. 

**Figure 8:**
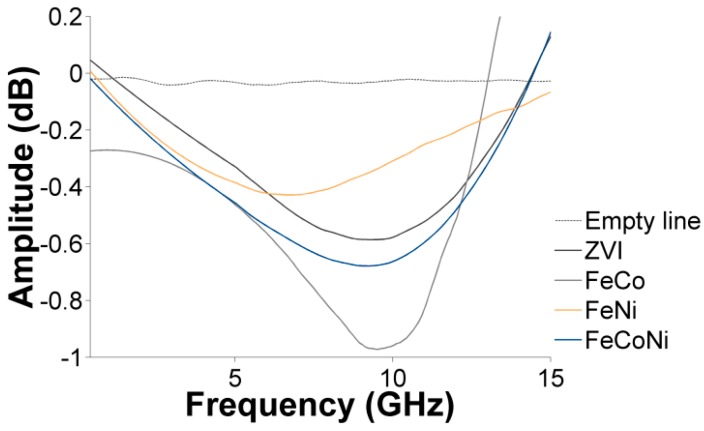
FMR (ferromagnetic resonance) absorption processes in the absence of an applied magnetic field for the different nanocomposites. A loading rate of 50 wt.% was aimed at in all cases.

**Figure 9:**
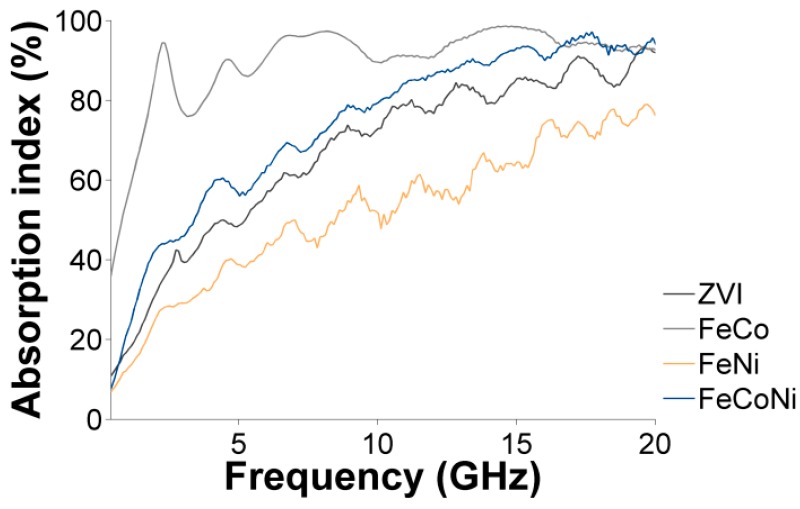
The percentage absorption rate of the incoming microwave by the different nanocomposites. A loading rate of 50 wt.% was aimed at in all cases.

The magnetic content of the nanocomposite samples, represented by the saturation magnetization, also impacted the intensity of the absorption around the natural FMR frequency (see Figure 8) and over the whole frequency range (see Figure 9). In both cases, the absorption intensity decreased in the order FeCo > FeNiCo > ZVI > FeNi, further demonstrating the relationship between the magnetic content of the nanocomposites and their microwave absorption behavior. The overall microwave absorption rate, *A*, shown in Figure 9, was calculated using the measured transmission (*S_21_*) and reflection (*S_11_*) scattering parameters and the relationship *A =* 1 *− |S*_11_*|*^2^ *− |S*_21_*|*^2^.

Additionally, the *S_11_* curves obtained for the different nanocomposites (see SM, Figure S15) showed that the reflection rate of the incoming microwave was low throughout the analyzed frequency range, roughly below −10 dB. Consequently, the incoming wave could penetrate the material throughout the investigated frequency range. The observed *A* rates were then mostly due to attenuation of the wave (lowered *S_21_*), produced by both the FMR effect and dielectric losses produced by the nanocarbon support. Indeed, the electric component of the incoming microwave-induced electrical currents within the conductive rGO sheets. Energetic loss related to the conduction of such electrical currents occurs using electrical resistance and heat generation. This phenomenon is known as Ohmic conduction and is the principal source of dielectric loss in the frequency range under study [77,78]. 

These results proved that these materials were suitable to produce EMI shielding solutions based on the effective absorption of the incoming microwaves, achieved in a much wider frequency range compared to similar absorber solutions [29,79]. In the case of the latter, low reflection is obtained in a very narrow frequency range so that the incoming wave can only penetrate and be absorbed in such a narrow band, limiting possible applications. Our materials overcame these limitations and showed the tailorable EMI potential obtained by the appropriate choice of nano-scaled materials.

## 4. Discussion

New types of microwave absorber nanocomposites were synthesized using the Pechini sol-gel method. Using this synthetic technique, reduced graphene oxide was decorated with ZVI@maghemite or equimolar binary and ternary Fe/Co/Ni alloys@carbon core@shell nanoparticles.

Certain synthetic parameters, such as the molar citric acid to ethylene glycol ratio (CA:EG) and the molar citric acid to the metal ratio (CA:M), were studied to determine their influence on the deposited NPs spatial distribution and size. It was found that the deposited NPs distributed more homogeneously over the nanocarbon surface when ethylene glycol and citric acid were used in similar quantities (1:1 and 1:1.5 ratios). These lower CA:EG ratios also created smaller NPs with narrower size distributions. Nonetheless, under the same synthetic conditions, ZVI NPs presented a monomodal size distribution, while bimodal distributions were observed in the three different types of alloy NPs. 

Core-shell structures were observed in the four different nanocomposites produced with our synthetic strategy. Nonetheless, their composition varied. Indeed, in the case of metallic iron, the shell was found to be composed of maghemite (*γ*-Fe_2_O_3_), while graphitic carbon was found surrounding the different alloy NPs. This result ensured that the deposited alloy NPs would be protected against air-driven oxidation processes. 

The magnetic properties of the different nanocomposites were also studied. All nanocomposites were found to present a weak ferromagnetic behavior at room temperature. Other properties, such as saturation magnetization, the intrinsic coercivity, and the remanent magnetization, varied according to the chemical nature of the deposited NPs. 

The electromagnetic properties of the different nanocomposites were studied in the microwave range (0.4 MHz to 20 GHz). An FMR absorption process was identified in all samples. Furthermore, a direct relationship between the nanocomposites’ natural FMR frequency and their composition-dependent saturation magnetization was found. To the best of our knowledge, this is the first time that such a relationship is determined for this type of nanocomposites. Based on these results, it appears that a wide library of alloy nanocomposites presenting different absorption frequencies could easily be created. Indeed, the alloy NPs Fe, Co, and/or Ni ratios could simply and reproducibly be varied using this synthetic technique to tune their properties. 

The microwave absorption efficiency of these new materials was also studied. It was shown that by using the same loading, absorption could be tuned from 60% to 100% by varying the nature of the metallic particles grafted onto rGO. Varying the microwave absorption rate allows designing compact impedance loads of specific values using the deposition of such nanocomposite samples on planar microwave circuits. Furthermore, the microwave absorption intensity can be varied by simply modifying the amount of metallic precursor engaged in the synthesis or the composition of the deposited NPs. Accordingly, these materials could be directly exploited to produce easily tunable, highly-efficient wideband microwave absorbers. 

## Figures and Tables

**Table 1:** ZVI (zero-valent iron) NPs (nanoparticles) size distributions obtained using different CA:M (citric acid to metal precursor) and CA:EG (citric acid to ethylene glycol) ratios.

	CA:M Ratio	
CA:EG Ratio	3:1	6:1
1:1	16.8 ± 6.5 nm	14.8 ± 4.7 nm
1:1.5	17.9 ± 8.1 nm	14.3 ± 7.2 nm
1:11	18.3 ± 9.6 nm	19.7 ± 10.4 nm

**Table 2:** ^57^Fe Mössbauer parameters for ZVI and Fe/Co/Ni alloy nanoparticles deposited on rGO (reduced graphene oxide).

Nanocomposite	T	*δ*	*ε*, *ΔEQ*	*B_hf_*	*Г/2*	Relative Area	Sites
	[K]	[mm/s]	[mm/s]	[Tesla]	[mm/s]	[%]	
ZVI@rGO	77	0.05(1)	–	–	0.18 *	4	*γ*-Fe (fcc)
		0.11(1)	0	34.1	0.21(1)	86	*α*-Fe (bcc)
		0.43(1)	0	39	0.28 *	10	*γ*-Fe_2_O_3_
FeCo@rGO	77	0.12(1)	0	34.6	0.22(1)	100	FeCo alloy
FeNi@rGO	77	0.16(1)	0.35(1)	–	0.15 *	6	superparamagnetic phase
		0.027(1) 0.06(2)	−0.018(1) 0.01(1)	34.1 31.5	0.20(1) 0.29(1)	24 70	FeNi (fcc) alloy
FeCoNi@rGO	77	0.35(1)	−0.07(1)	21.2	0.40(1)	30	Fe carbide
		0.15(1)	−0.05(1)	32	0.17(1)	70	FeCoNi (fcc) alloy

(1) *δ*: isomer shift (with respect to *α*-Fe at r.t.); *ε*, *ΔEQ*: quadrupole splitting; *Γ/2*: half-width at half maximum. * Fixed parameters.

**Table 3:** Elemental mass composition (wt.%) for the different alloy nanocomposites determined by EDX and ICP.

	EDX			ICP		
Alloy NPs	% Fe	% Co	% Ni	% Fe	% Co	% Ni
FeCo	47 ± 4	53 ± 6	–	49	51	–
FeNi	48 ± 3	–	52 ± 3	52	–	48
FeCoNi	30 ± 8	36 ± 5	34 ± 6	32	34	34

**Table 4:** Magnetic properties of the ZVI and Fe/Co/Ni alloy nanocomposites. A loading rate of 50 wt.% was aimed at in all cases.

Nanocomposite	SPL [63,64] (nm)	% NPs < SPL	*M_s_* 300 K (emu/g)	*M_r_* 300 K (emu/g)	*H_c_* 300 K (Oe)
ZVI@rGO	~14	75%	91	20	265
FeCo@rGO	~22	90%	205	24	141
FeNi@rGO	~30	84%	124	11	78
FeCoNi@rGO	N.D.	–	187	4	21

## Supplementary Materials

The following are available online at https://www.mdpi.com/2079-4991/9/9/1196/s1, Section S1: Reactants specifications; Section S2: Characterization techniques; Section S3: Supplementary information; Figure S1: Schematic representation of the Pechini synthesis; Figure S2: Representative TEM images of ZVI@rGO nanocomposites synthesized using different combinations of CA:EG and CA:M ratios; Figure S3: Schematic representation of the influence of the citric acid to ethylene glycol or metal molar ratios; Table S1: Fe/C mass loading rate (%LR) obtained using different CA:M and CA:EG ratios determined by TGA; Figure S4: Thermograms for the ZVI@rGO nanocomposites obtained with 20%, 30%, 40%, and 60% ZVI NPs wt. %LRs; Table S2: C_1s_ components % atomic concentration as obtained by XPS for a ZVI@GO nanocomposite and commercial samples of GO and rGO; Figure S5: C1s peak decomposition as obtained by XPS for a ZVI@GO nanocomposite and commercial samples of GO and rGO; Figure S6: ^57^Fe Mössbauer spectra at 77 K of ZVI and Fe/Co/Ni alloy nanoparticles deposited on rGO; Figure S7: O_1s_ peak decomposition obtained by XPS for a typical ZVI@GO nanocomposite; Figure S8: Typical TEM images of the FeCo@rGO, FeNi@rGO, and FeCoNi@rGO nanocomposites prepared with 1:1.5 CA:M and 3:1 CA:EG ratios; Figure S9: HRTEM images illustrating core-shell structures found in FeCo, FeNi, and FeCoNi nanocomposites; Figure S10: ToF-SIMS spectra of the FeCo@rGO (a), FeNi@rGO (b), and FeCoNi@rGO (c) nanocomposites showing the presence of binary metal oxides moieties; Figure S11: ZVI and Fe/Co/Ni alloy nanocomposites *M_s_*, *H_c,_* and *M_r_* values obtained from full magnetization curves recorded against an applied magnetic field at 300K; Figure S12: Coplanar waveguide (CPW) transmission (*S_21_*) measurements and difference between *S_21_* and *S_12_* parameters for a ZVI@rGO nanocomposite; Figure S13: Dispersion relation between the applied magnetic field and the FMR absorption frequency for all the synthetized nanocomposites; Figure S14: Results for the chain matrix formalism simulation of (a) zero-field FMR absorption (S21 parameter) of a *α*-Fe@*γ*-Fe_2_O_3_ core@shell NP; and (b) the real (*μ’*) and imaginary (*μ’’*) components of the core and shell’s relative permeability, *μ_r_*; Figure S15: Coplanar waveguide (CPW) reflection (*S_11_*) measurements for the different MNPs@rGO nanocomposites at null magnetic field.
