# Supplementary material for: Highly Efficient Wideband Microwave Absorbers Based on Zero-Valent Fe@γ-Fe2O3 and Fe/Co/Ni Carbon-Protected Alloy Nanoparticles Supported on Reduced Graphene Oxide
Source: Nanomaterials (Basel). 2019 Aug 25;9(9):1196. doi: 10.3390/nano9091196 (PMC6780371; doi:10.3390/nano9091196)

Highly Efficient Wideband Microwave Absorbers Based on Zero-Valent Fe@γ-Fe_2O_3 and Fe/Co/Ni Carbon-Protected Alloy Nanoparticles Supported on Reduced Graphene Oxide

Francisco Mederos-Henry [1], Julien Mahin [1,2], Benoit P. Pichon [3], Marinela M. Dîrtu [1,4], Yann Garcia [1], Arnaud Delcorte [5], Christian Bailly [5], Isabelle Huynen [6] and Sophie Hermans [1,*]

1 Institute of Condensed Matter and Nanosciences (IMCN), Division of Molecules, Solids and Reactivity (MOST), Place Louis Pasteur 1, Université catholique de Louvain, B-1348 Louvain-la-Neuve, Belgium
2 Department of Chemical Engineering and Biotechnology, University of Cambridge, Phillipa Fawcett Drive, West Cambridge Site, Cambridge CB3 0AS, UK
3 Institut de Physique et Chimie des Matériaux de Strasbourg, CNRS, Université de Strasbourg, UMR 7504, F-67000 Strasbourg, France
4 Faculty of Electrical Engineering and Computer Science & MANSiD Research Center, Stefan cel Mare University, 720229 Suceava, Romania
5 Institute of Condensed Matter and Nanosciences (IMCN), Division of Bio and Soft Matter (BSMA), Croix du Sud 1, Université catholique de Louvain, B-1348 Louvain-la-Neuve, Belgium
6 Institute of Information and Communication Technologies, Electronics and Applied Mathematics (ICTEAM), Place du Levant 3, Université catholique de Louvain, B-1348 Louvain-la-Neuve, Belgium
* Correspondence: sophie.hermans@uclouvain.be; Tel.: +32-1047-2810

SECTION S1 – Reactants specifications

Nanocarbon support

Name	Company	Location
Graphene oxide (GO)	Nanoinnova Technologies SL	Toledo, Spain

Other reactants

Name	Purity/Grade	Supplier
Ethanol	Technisolv	VWR, Belgium
Iron (III) nitrate nonahydrate	99+%	Acros Organics, Merelbeke, Belgium
Cobalt (II) nitrate hexahydrate	For analysis	Acros Organics, Merelbeke, Belgium
Nickel (II) nitrate hexahydrate	97+%	Sigma Aldrich, Darmstadt, Germany
Citric acid	For analysis	Merck, Overijse, Belgium
Ethylene glycol	Spectrophotometric grade, 99+%	Alfa Aesar, Kandel, Germany

SECTION S2 – Characterization techniques

Inductively coupled plasma atomic emission spectroscopy (ICP-AES). Samples (~25 mg) of Fe/Co/Ni alloy NPs deposited onto rGO were carbonized at 550°C. The leftover ashes were dissolved in 0.05 mL of *aqua regia* and then diluted 1000x. The resulting solutions were measured with an ICAP 6500 Thermoscientific spectrophotometer.

^{57}Fe Mössbauer spectroscopy. Samples were sealed in aluminum foil and mounted in a nitrogen Oxford bath cryostat. ^{57}Fe Mössbauer spectra were recorded at 77 K and room temperature (R.T.) in transmission geometry using a conventional Mössbauer spectrometer equipped with a ^{57}Co(Rh) radioactive source operating at room temperature. The spectra were fitted to the sum of Lorentzians by a least-squares refinement using Recoil 1.05 Mössbauer Analysis Software[1]. All isomer shifts refer to -Fe at room temperature.

Powder X-ray diffraction (XRPD). Samples were introduced into 0.5 mm thin-walled glass capillaries (Hilgenberg GmbH, Germany), mounted on a goniometer head and kept at 200 mm distance from the detector. Diffractograms were then collected at room temperature using a MAR345 diffractometer (MarResearch GmbH), a Mo-Kα (0.71073 Å) anode and a XENOCS focusing mirror. The obtained 2D diffractograms were azimuthally integrated using the Fit2D software, calibrated with a LaB6 standard (NIST 660b Standard).

Scanning electron microscopy with coupled energy-dispersive X-ray spectroscopy (SEM-EDX). Samples prepared for TEM analysis were also used for SEM imaging. The

TEM grids were mounted on double-face adhesive carbon tape adhered to an aluminum sample holder. These were analyzed without further preparation in a FEGSEM Ultra55 instrument (Carl Zeiss) equipped with an Oxford Inca EDX system (Oxford Instruments). Images were acquired with the SmartSEM software (Carl Zeiss, Germany) at different acceleration voltages ranging between 3 keV and 15 keV, using InLens and SE2 detectors. X-ray fluorescence spectra were collected at 15 keV using the ESPRIT software (Bruker, Germany).

Scattering matrix (S-parameters) measurements. The EM measurements were performed directly on the synthesized powders following a novel vector network analyzer (VNA) methodology reported elsewhere[2,3]. Nanocomposites samples were mixed with ethanol and introduced into rectangular cavities drilled in the two slots of a coplanar line etched on a RO4350B™ substrate, pressing with a spatula to compact the sample slurry. Once completely filled, the as-prepared line was dried under vacuum at 25 ºC for 2 hours. The loaded line was characterized under DC magnetic field applied perpendicularly using a NTM 10400M-260 electromagnet supplying magnetic field values ranging from 0 to 9 kOersteds (kOe). The sample was placed in the gap of the electromagnet and connected to one end of a set of long coaxial cables using a pair of Anritsu 36801K right angle launchers. The other coaxial cables ends were connected to the ports of a 12-term calibrated Anritsu 37369A 40 GHz vector network analyzer (VNA), placed away from the electromagnet's intense DC magnetic field. Data acquisition was performed using Labview and the obtained measurements

were normalized using a standardized Matlab background subtraction technique, in order to remove spurious losses induced by connectors and substrate.

Superconducting Quantum Interference Device (SQUID) magnetometer. Magnetization measurements were performed using a Quantum Design MPMS-XL5 SQUID magnetometer without any further sample preparation. Magnetization curves as a function of an applied magnetic field (M(H) curve) were measured at 300 K after applying a degaussing procedure. The magnetization was then measured at constant temperature by sweeping back and forth the magnetic field between +7 T and -7 T.

Time of flight secondary ion mass spectrometry (ToF-SIMS). Sample powders were manually pressed with a spatula onto adhesive parts of Post-it® papers and analysed without further preparation using a IONTOF TOF.SIMS instrument (IONTOF, GmbH, Münster, Germany). A pulsed Bi+ metal ion source produced a primary beam using an acceleration voltage of 30 kV. An AC target current of 2 pA with a bunched pulse width lower than 1 ns was used. Both positive and negative secondary ion species were analysed. For spectra acquisition, a raster of 128 x 128 data points over an area of 200 x 200 μm^2 was employed. The total primary ion beam dose for each analysed area was always kept below $2x10^{12}$ $ions.cm^{-2}$. Lateral resolution of ~3 μm and mass resolution $m/\Delta m > 5500$ at 29 m/z were maintained for positive and negative spectra acquisition. Charge compensation was done with an interlaced electron flood gun (E_k = 20 eV). All data analyses were carried out using the software supplied by the instrument manufacturer, SurfaceLab (v.6.5).

Transmission electron microscopy (TEM). Samples were dispersed in hexane or ethanol by sonication. Three drops of the supernatant were then deposited onto a holey carbon film supported on a copper grid (C-flat, Protochips, USA), and left to dry, overnight, at room temperature under vacuum. TEM images were obtained on a LEO 922 OMEGA Energy Filter Transmission Electron Microscope operating at 120 kV. Particle sizes were measured with the AnalySIS Auto 5.0 software (Olympus Soft Imaging Solutions GmbH, Germany) on at least 100 particles per sample, as recorded on high-magnification TEM images. High resolution TEM (HRTEM), EDX, and electron diffraction (ED) were performed with a JEOL ARM200F microscope operating at 200 kV.

Thermogravimetric analysis (TGA). ZVI@rGO nanocomposite samples (5-10 mg) were placed in alumina containers and introduced in a Mettler Toledo TGA/SDTA 851e instrument. Thermograms were recorded up to 900 ºC using a 10 °C/min heating rate under an air flux. The samples were recovered after heating and analyzed by XRD as described above, identifying in all cases a hematite (Fe_2O_3) phase. TGA data treatment required for the calculation of the Fe/NcS loading rates was based on the formula weight of this particular iron oxide phase. Corrections for residual non-carbonaceous impurities present in GO were also included in these calculations.

X-Ray photoelectron spectroscopy (XPS). A few milligrams of each sample were deposited on a double-sided adhesive tape clung onto a brass cup, and then introduced onto a Macor® carousel. The analyses were performed on a SSX 100/206 photoelectron spectrometer from Surface Science Instruments (USA) equipped with a

monochromatized micro focused Al X-ray source (powered at 20 mA and 10 kV). The pressure in the analysis chamber was around 10^{-6} Pa. A flood gun set at 8 eV and a Ni grid placed 3 mm above the sample surface were used for charge stabilization. The C-(C,H) component of the carbon C1s peak was fixed to 284.8 eV to set the binding energy scale. Data treatment was performed with the CasaXPS program (Casa Software Ltd, UK). Spectra were decomposed with the least squares fitting routine provided by the software with a Gaussian/Lorentzian (85/15) product function and after subtraction of a Shirley-type baseline[4]. Molar fractions were calculated using peak areas normalized on the basis of acquisition parameters and sensitivity factors provided by the manufacturer.

Bibliography

1. K. Lagarec and D. G. Rancourt. Mössbauer Spectral Analysis Software for Windows 1.0. Department of Physics, University of Ottawa, Canada, 1998.
2. F. Mederos-Henry, S. Hermans and I. Huynen, *Microw. Opt. Technol. Lett.*, 2017, **59**, 2330–2335.
3. F. Mederos-Henry, S. Hermans and I. Huynen, *J. Nanomater.*, 2019, Article ID 3280461.
4. D.A. Shirley Phys. Rev., 1972, **B5**, 4709-4714.

SECTION S3 – Supplementary information

Figure S1- Schematic representation of the Pechini synthesis. 'M' stands for the complexated metal, the brown triangles for the citrate ion and gray lines for ethylene glycol. Adapted from Danks *et al.* [22].

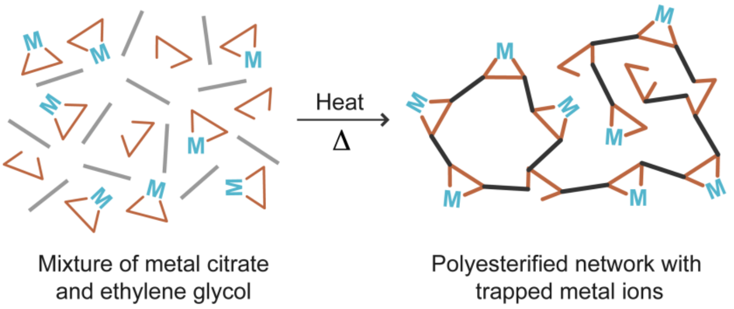

Figure S2 - Representative TEM images of ZVI@rGO nanocomposites synthesized using different combinations of CA:EG and CA:M ratios.

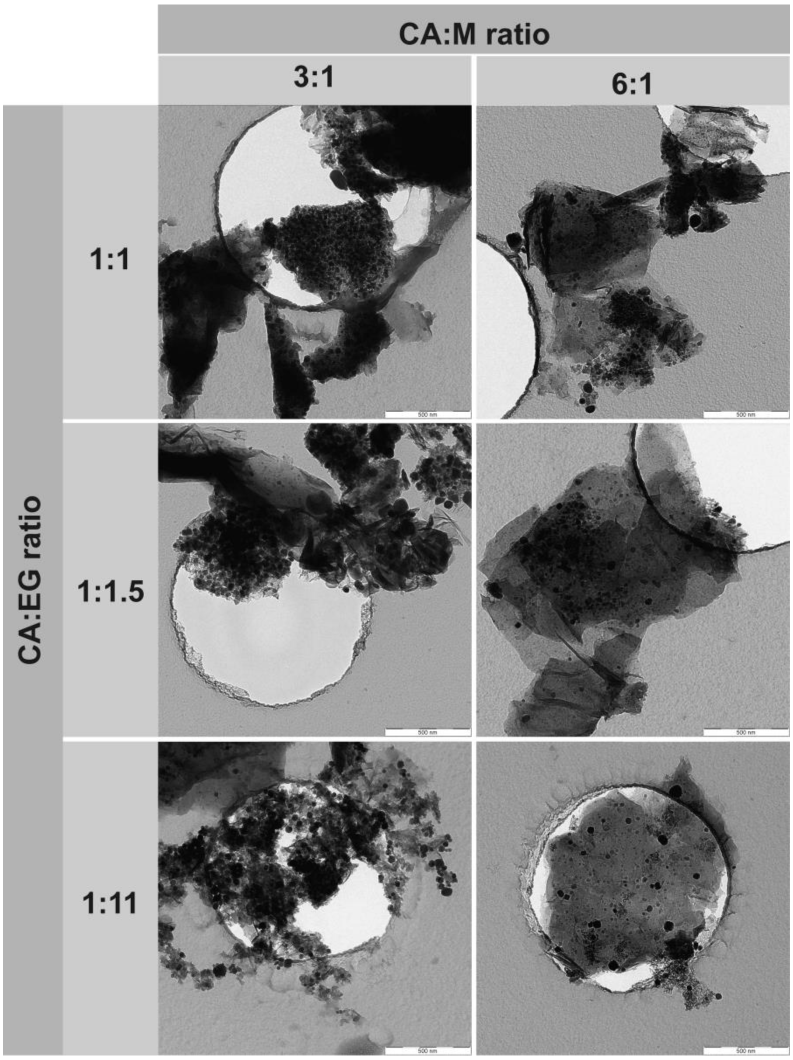

Figure S3 – Schematic representation of the influence of the citric acid (CA) to ethylene glycol (EG) or metal (M) molar ratios (top and bottom figures, respectively). 'M' stands for the complexated metal, the brown triangles for the citrate ion and gray lines for ethylene glycol.

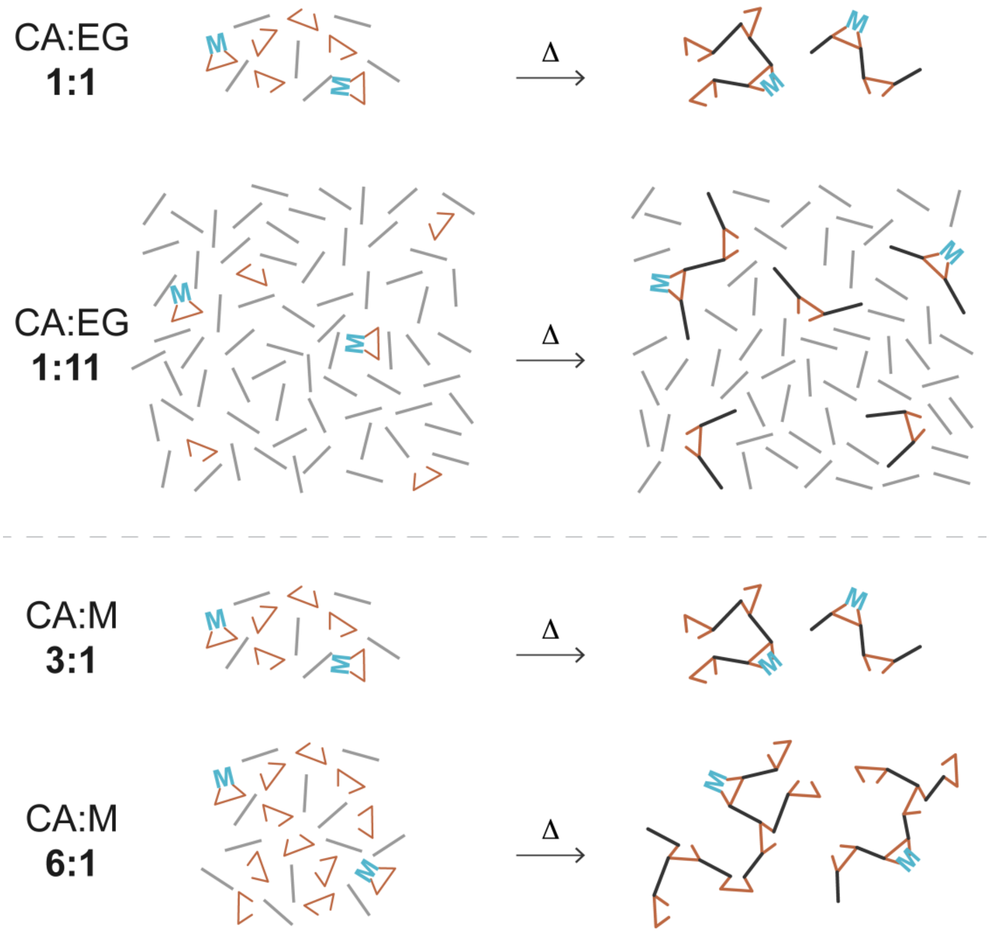

Table S1 - Fe/C mass loading rate (%LR) obtained using different CA:M and CA:EG ratios determined by TGA. A 50% LR was aimed at in all cases.

	**CA:M ratio**	
**CA:EG ratio**	**3:1**	**6:1**
**1:1**	48	48
**1:1.5**	44	41
**1:11**	43	38

Figure S4- Thermograms for the ZVI@rGO nanocomposites obtained with 20%, 30%, 40% and 60% ZVI NPs wt. %LRs.

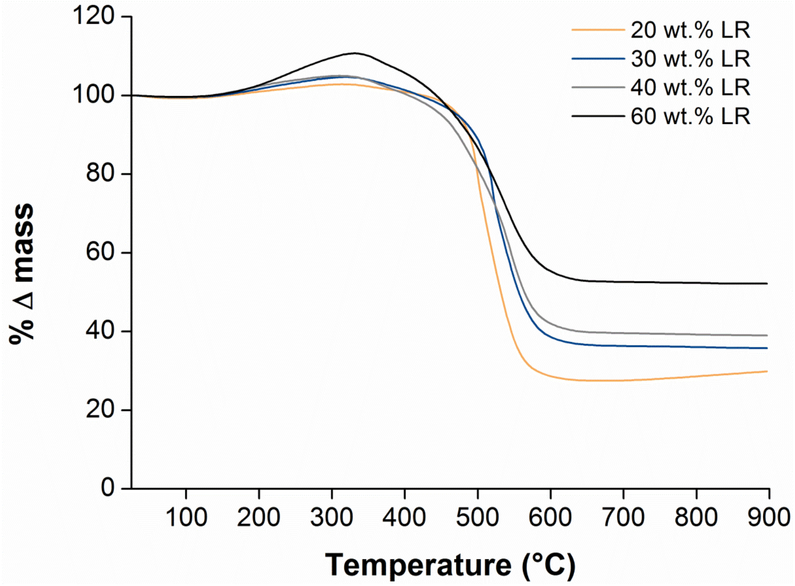

Table S2- C_{1s} components % atomic concentrations as obtained by XPS for a ZVI@GO nanocomposite and commercial samples of GO and rGO supplied by Nanoinnova Technologies SL

	**C_{1s} components (% atomic concentration)**				
**NcS**	C_{arom}	C-(C,H)	C-O	C=O, O-C-O	(C=O)-OH
ZVI@GO	27.6	54.6	12.3	4.0	1.5
GO *	6.5	36.6	7.5	41.6	7.8
rGO *	87.3	3.3	5.9	3.0	0.5

* *commercial samples*

Figure S5- C1s peak decomposition obtained by XPS for a ZVI@GO nanocomposite and commercial samples of GO and rGO supplied by Nanoinnova Technologies SL

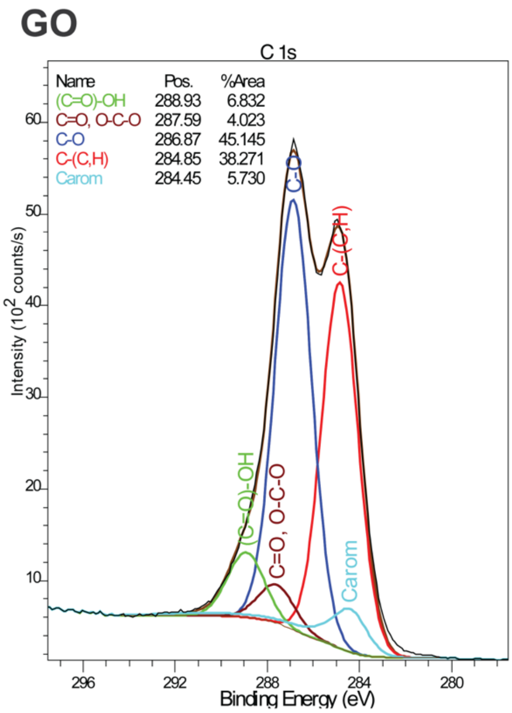

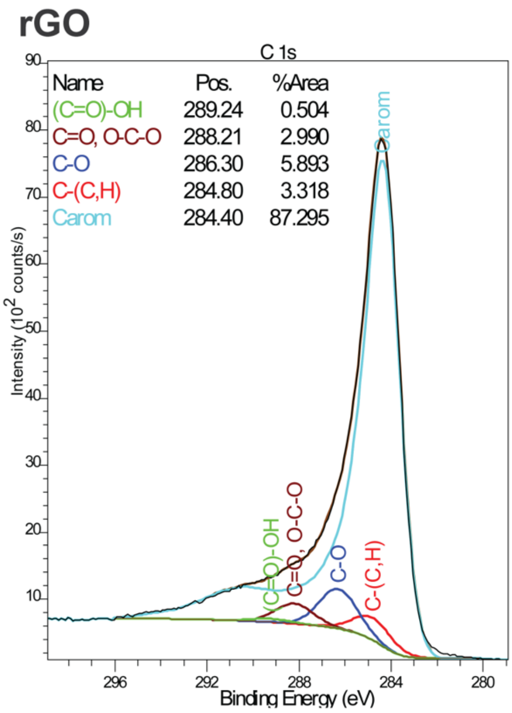

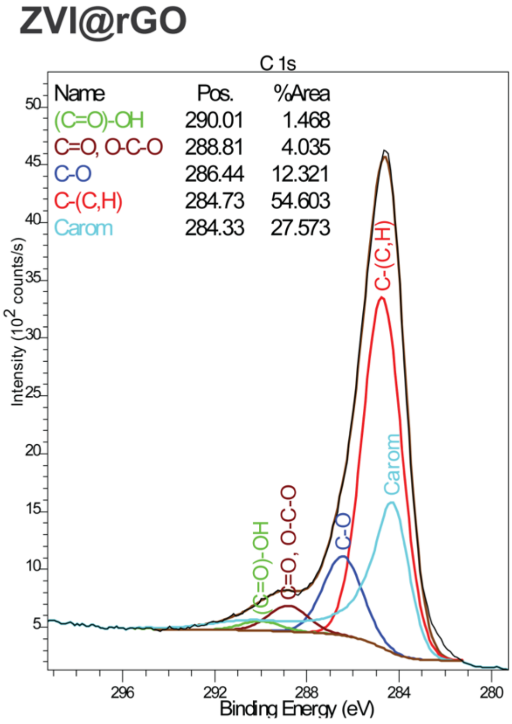

ZVI@rGO nanocomposite synthetized using a 1:1.5 citric acid:ethylene glycol ratio, a 3:1 citric acid:metal ratio, and a 50% intended Fe/NcS loading rate (% LR).

Figure S6 - ^{57}Fe Mössbauer spectra at 77K of ZVI and Fe/Co/Ni alloy nanoparticles deposited on rGO: **(a)** ZVI@rGO; **(b)** FeCo@rGO; **(c)** FeNi@rGO; and **(d)** FeCoNi@rGO.

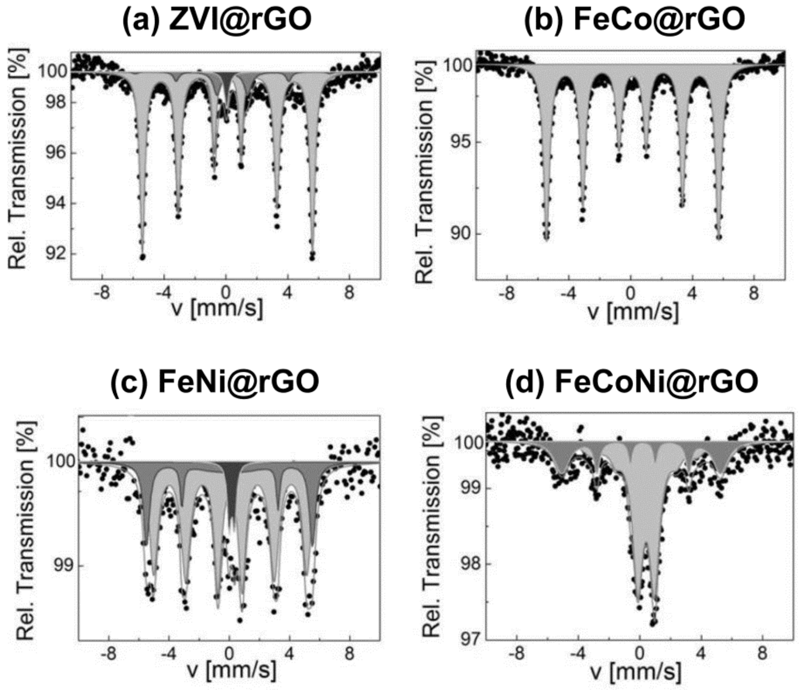

Figure S7 - O_{1s} peak decomposition obtained by XPS for a typical ZVI@GO nanocomposite prepared with 1:1.5 CA:M and 3:1 CA:EG ratios.

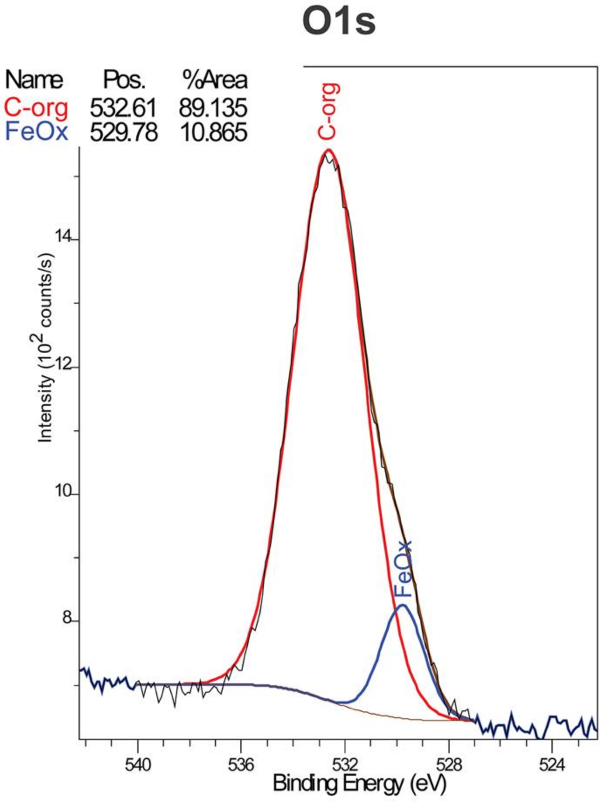

Figure S8 - Typical TEM images of the FeCo@rGO, FeNi@rGO and FeCoNi@rGO nanocomposites prepared with 1:1.5 CA:M and 3:1 CA:EG ratios.

FeCo@rGO

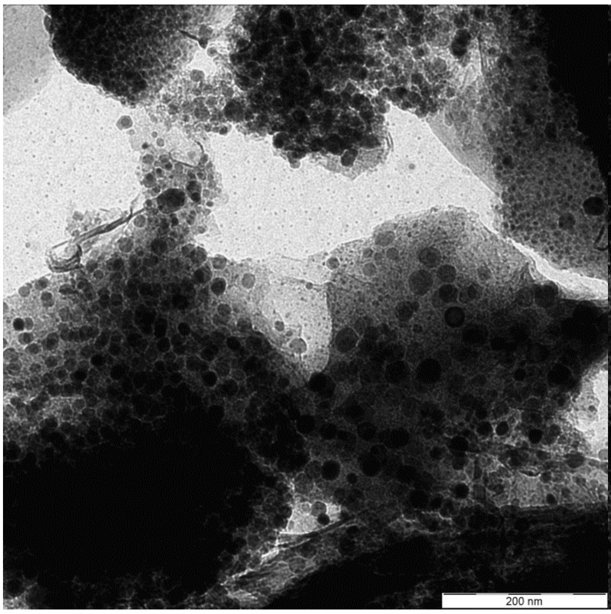

FeNi@rGO

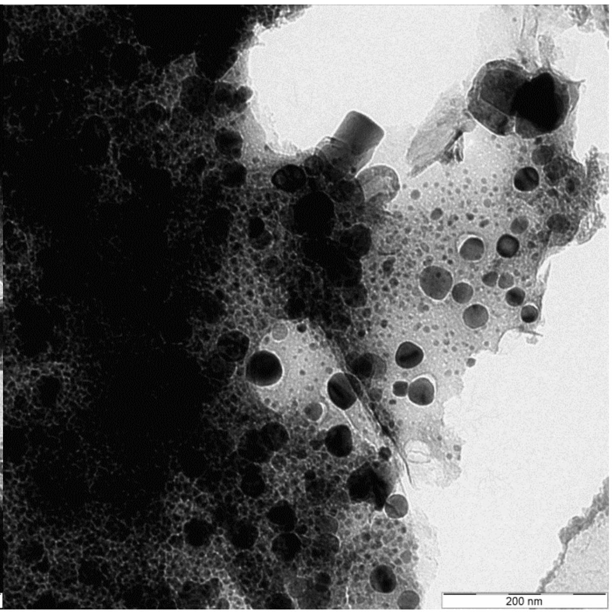

FeCoNi@rGO

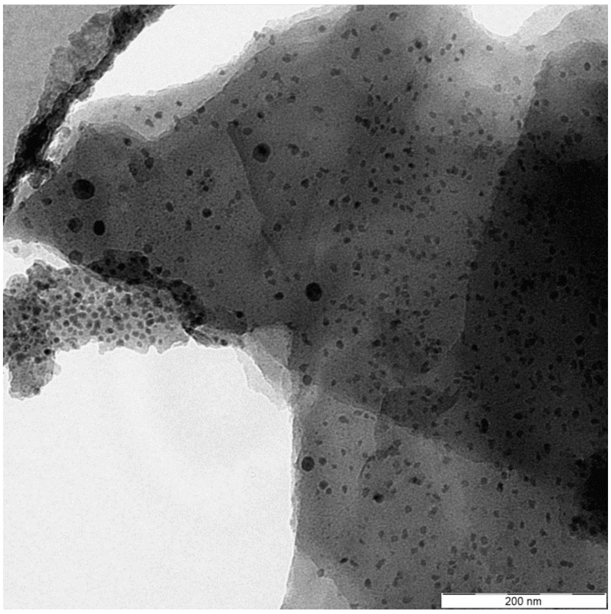

Figure S9 - HRTEM images illustrating core-shell structures found in FeCo (top, left), FeNi (top, right) and FeCoNi (bottom) nanocomposites.

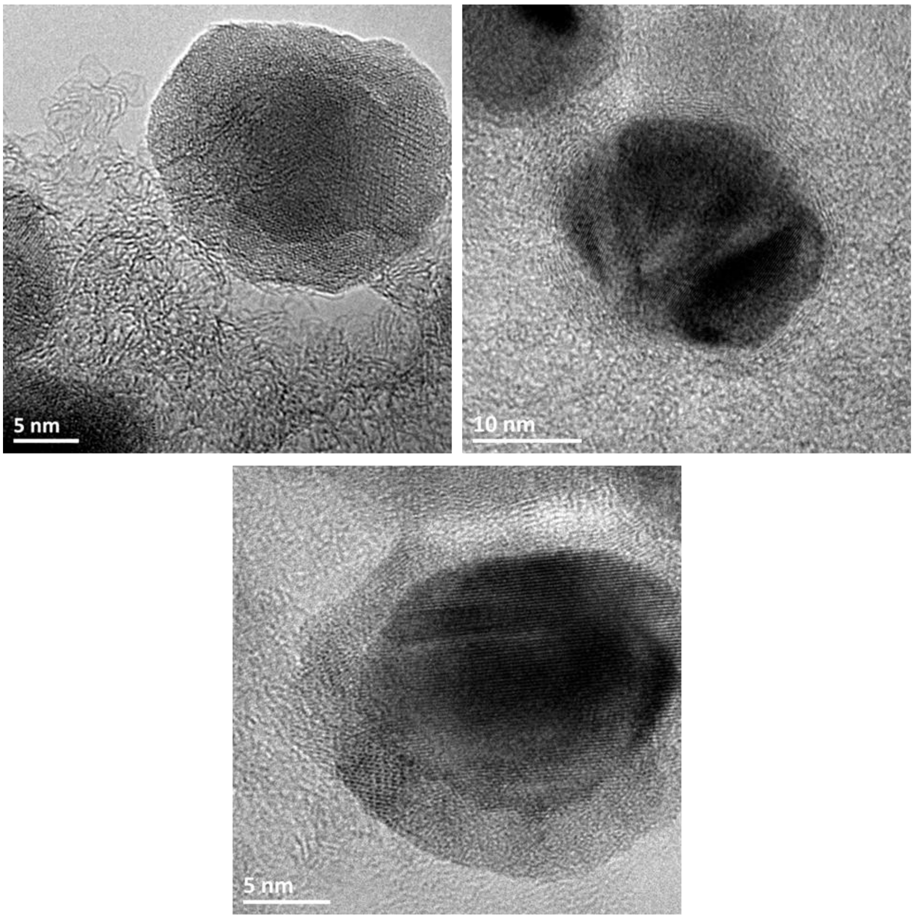

Figure S10 - ToF-SIMS spectra of the FeCo@rGO (a), FeNi@rGO (b) and FeCoNi@rGO (c) nanocomposites showing the presence of binary metal oxides moieties.

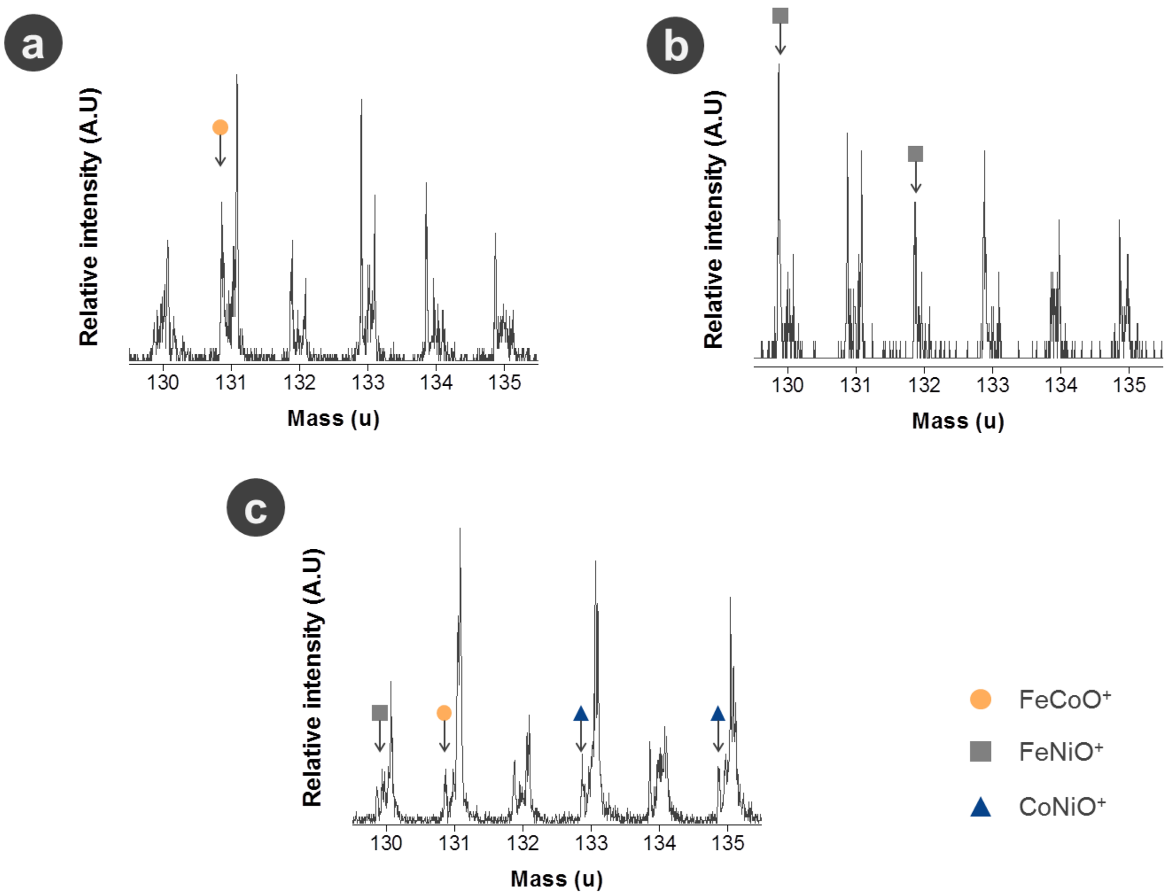

Species	Mass (u)	Relative abundance
**$FeCoO^+$**	130.8630	92%
**$FeNiO^+$**	129.8650	62%
	131.8610	24%
**$CoNiO^+$**	132.8640	68%
	134.8590	26%

Figure S11 - ZVI and Fe/Co/Ni alloy nanocomposites M_s (top), H_c and M_r values (bottom) obtained from full magnetization curves recorded against an applied magnetic field at 300K. A loading rate of 50 wt.% metal/NcS was aimed at in all cases

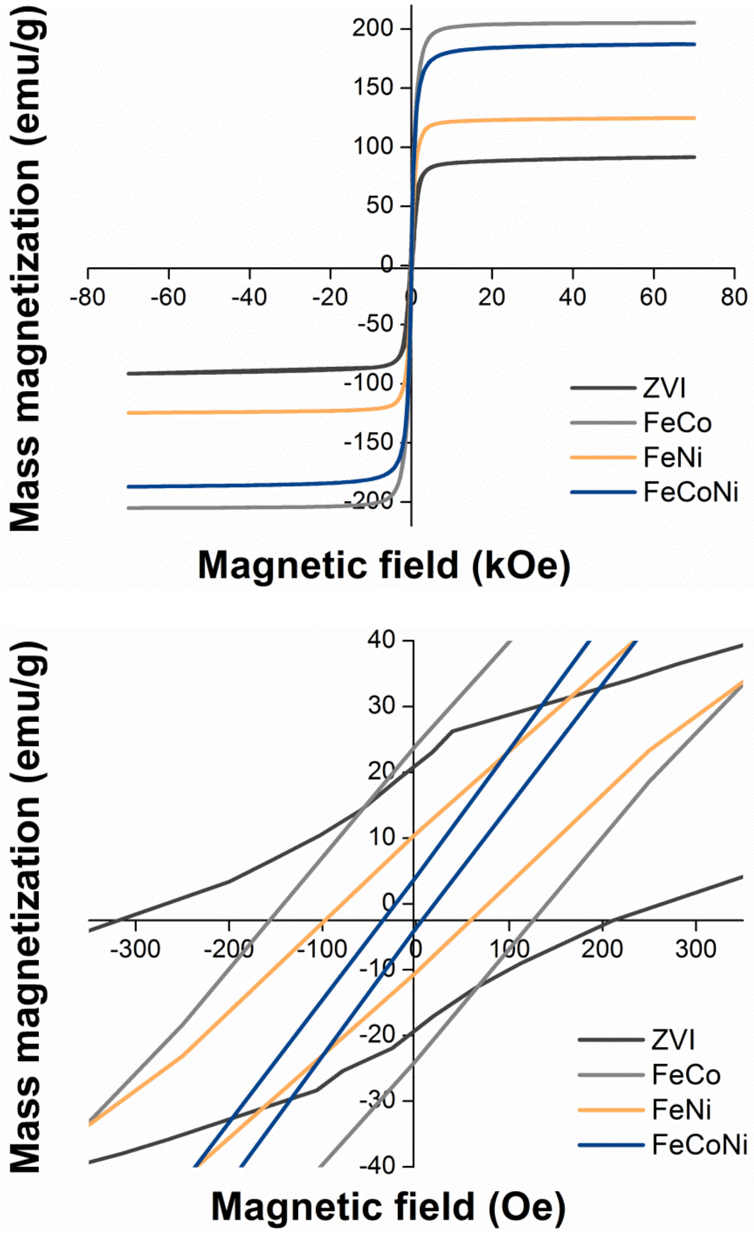

Figure S12 – Coplanar waveguide (CPW) transmission (S_{21}) measurements (top) and difference between S_{21} and S_{12} parameters (bottom) for a ZVI@rGO nanocomposite. Measurements were performed under DC magnetic field ranging from 0 to 5 kOe. The nanocomposite was synthesized using 1:1.5 CA:M and 3:1 CA:EG ratios, aiming a 50 wt.% Fe/NcS loading rate.

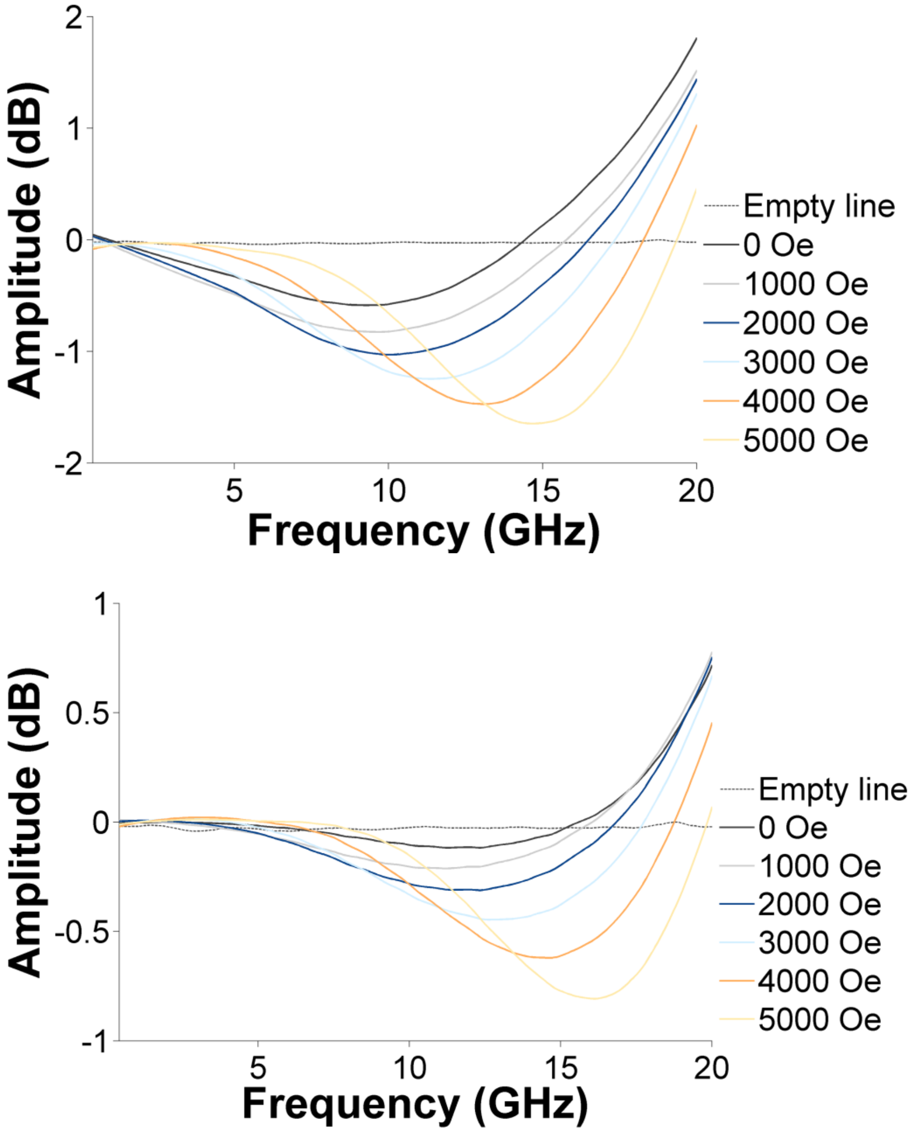

Figure S13 - Dispersion relation between the applied magnetic field and the FMR absorption frequency for all the synthetized nanocomposites. The filled circles represent the experimental data. The dotted lines are the linear regressions of such data.

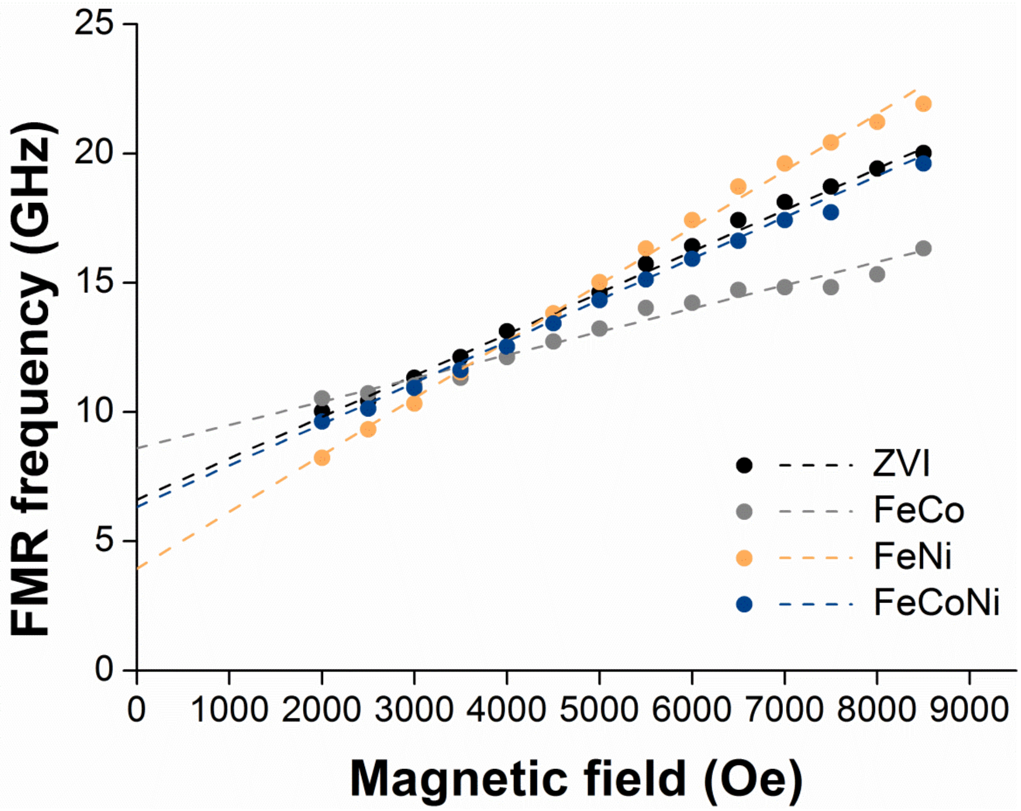

	ZVI@rGO	FeCo@rGO	FeNi@rGO	FeCoNi@rGO
**Natural FMR frequency (GHz)**	6.6	8.6	3.9	6.3

Figure S14 - Results for the chain matrix formalism simulation of (a) zero-field FMR absorption (*S_{21}* parameter) of a -Fe@ -Fe_2O_3 core@shell NP; and (b) the real (′) and imaginary (′′ components of the core and shell′s relative permeability, $_r$. The dimensions of the simulated NP are shown in (c) and are based on TEM observations of the ZVI@rGO nanocomposite produced using 6:1 CA:M and 1:1.5 CA:EG ratios.

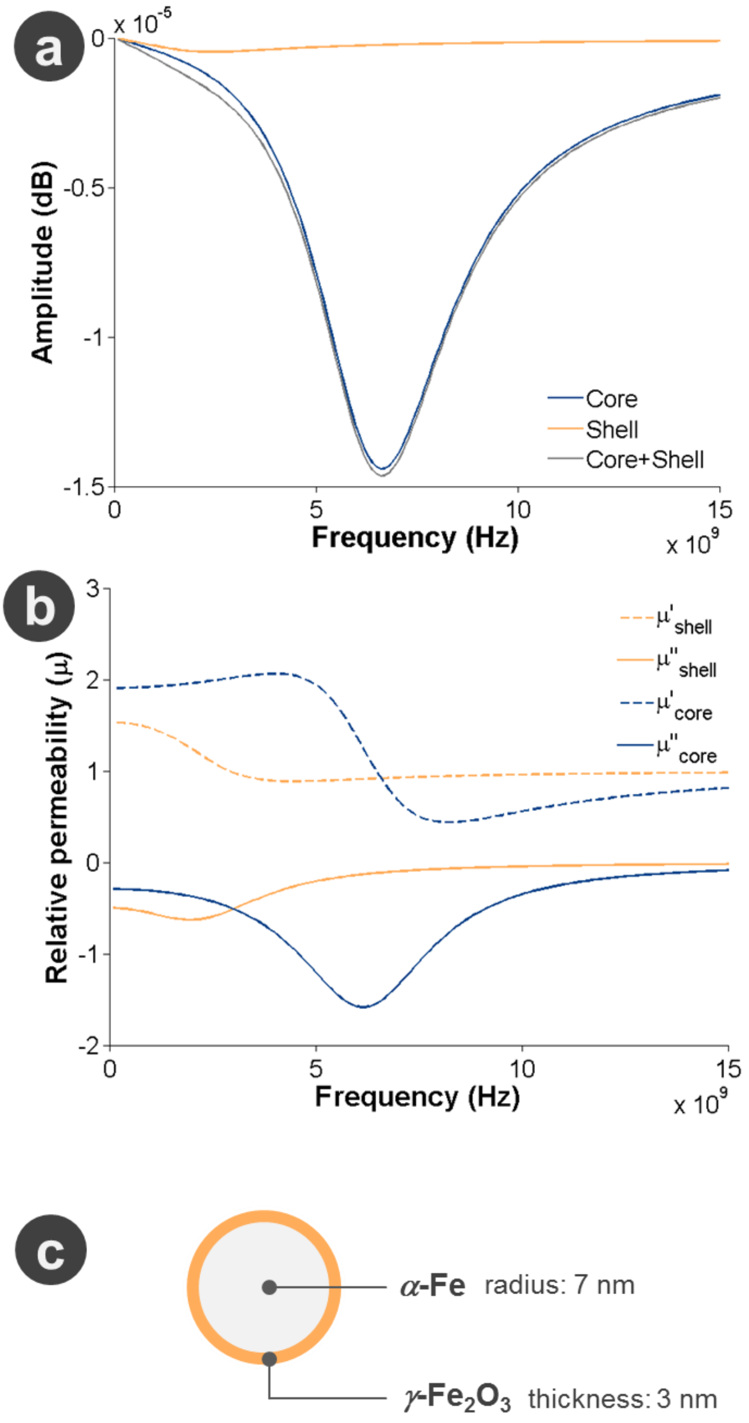

Figure S15 - Coplanar waveguide (CPW) reflexion (S11) measurements for the different MNPs@rGO nanocomposites at null magnetic field. All nanocomposites were synthesized using 1:1.5 CA:M and 3:1 CA:EG ratios, aiming a 50 wt.% loading rate.

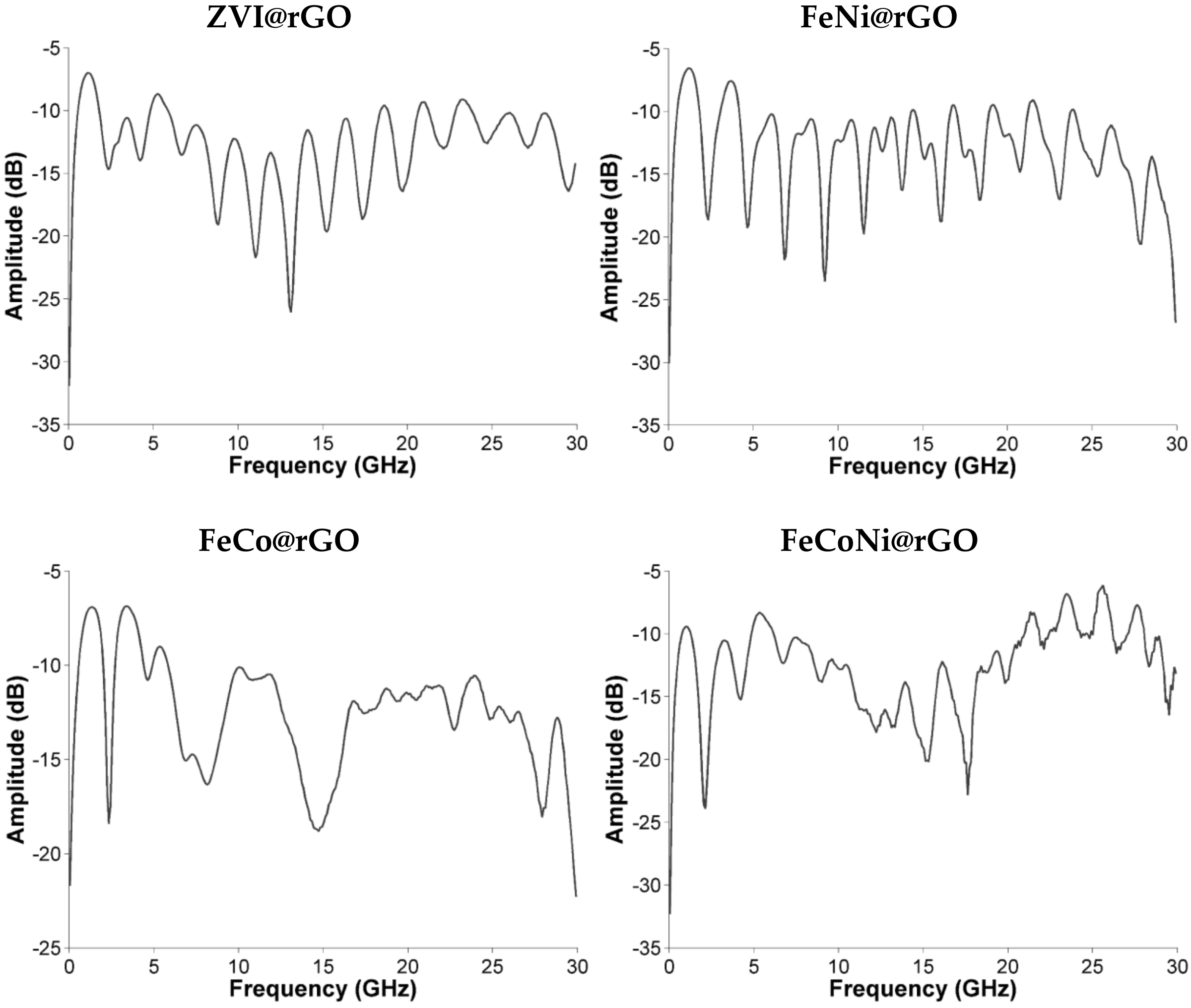

Supplement: Supplementary file 1 [file nanomaterials-09-01196-s001.pdf]
